# Are Genetic Modifiers the Answer to Different Responses to Hydroxyurea Treatment?—A Pharmacogenetic Study in Sickle Cell Anemia Angolan Children

**DOI:** 10.3390/ijms24108792

**Published:** 2023-05-15

**Authors:** Catarina Ginete, Mariana Delgadinho, Brígida Santos, Vera Pinto, Carina Silva, Armandina Miranda, Miguel Brito

**Affiliations:** 1 H&TRC—Health & Technology Research Center, ESTeSL—Escola Superior de Tecnologia da Saúde, Instituto Politécnico de Lisboa, 1990-096 Lisbon, Portugal; catarina.ginete@estesl.ipl.pt (C.G.); mariana.delgadinho@estesl.ipl.pt (M.D.); vgpinto@ciencias.ulisboa.pt (V.P.); carina.silva@estesl.ipl.pt (C.S.); 2 Centro de Investigação em Saúde de Angola (CISA), Bengo, Angola; santosbrigida@yahoo.com.br; 3 Hospital Pediátrico David Bernardino (HPDB), Luanda, Angola; 4 Centro de Estatística e Aplicações, Universidade de Lisboa (CEAUL), 1749-016 Lisbon, Portugal; 5 Instituto Nacional de Saúde Doutor Ricardo Jorge (INSA), 1649-016 Lisbon, Portugal; armandina.miranda@insa.min-saude.pt

**Keywords:** sickle cell anemia, hydroxyurea, pharmacogenetics, next-generation sequencing (NGS)

## Abstract

Sickle cell anemia (SCA) is an inherited disease affecting the hemoglobin that is particularly common in sub-Saharan Africa. Although monogenic, phenotypes are markedly heterogeneous in terms of severity and life span. Hydroxyurea is still the most common treatment for these patients, and the response to treatment is highly variable and seems to be an inherited trait. Therefore, identifying the variants that might predict hydroxyurea response is important for identifying patients who will have a poorer or non-response to treatment, and the ones that are more prone to suffer from severe side effects. In the present pharmacogenetic study, we analyzed the exons of 77 genes described in the literature as potentially associated with hydroxyurea metabolism in Angolan children treated with hydroxyurea and evaluated the drug response considering fetal hemoglobin levels, other hematological and biochemical parameters, hemolysis, number of vaso-occlusive crises and hospitalizations. Thirty variants were identified in 18 of those genes as possibly associated with drug response, five of them in gene DCHS2. Other polymorphisms in this gene were also associated with hematological, biochemical and clinical parameters. Further research examining the maximum tolerated dose and fixed dose with a larger sample size is necessary to corroborate these findings.

## 1. Introduction

Sickle cell anemia (SCA) is an inherited disease, where the presence of a homozygous mutation in the gene that encodes the hemoglobin subunit β (HBB, c.20T>A, p.Glu6Val) results in the presence of a defective hemoglobin that tends to polymerize under deoxygenated conditions, causing red blood cells (RBC) to be denser and rigid, and increasing blood viscosity. Although SCA is a monogenic disease, phenotypes are markedly heterogeneous in terms of severity and life span [1,2]. This disease is particularly common in sub-Saharan Africa, with around 75% of SCA births occurring in this region [3].

One important modulator of SCA severity is Fetal Hemoglobin (HbF), the most prevalent hemoglobin in the last two trimesters of gestation in humans, having an impact on the clinical and hematological features of this disease [4]. Several genetic modifiers have been associated with HbF levels and their persistence through adulthood, being the genetic loci that present the most important modifier variants: HBS1L-MYB intergenic region, BCL11A and the B-globin gene cluster [4,5]. In some cases, where stress-based erythropoiesis happens, the inherence of heterocellular hereditary persistence of HbF (HPFH) may lead to increases in HbF levels to 25%, which considerably improves SCA patients’ phenotypes [6].

Hydroxyurea (HU) was the first therapy approved for clinical use in sickle cell disease (SCD) patients by the US FDA (since 1998) and the European Medicines Agency (since 2007), and the only one approved until 2019 [7,8]. This drug, a ribonucleotide reductase inhibitor, was first considered an anti-neoplastic agent used to treat patients with myeloproliferative diseases. Treatment with HU in SCD patients has the purpose of inducing HbF (α_2_γ_2_), increasing the γ-globin gene expression and consequently reducing the proportion of adult hemoglobin (α_2_β_2_) [6].

By inducing HbF expression, HU reduces sickling and therefore vaso-occlusion events. It also reduces platelet, leukocyte and reticulocyte counts, lactate dehydrogenase (LDH), cell adhesion molecules and increases nitric oxide levels [9,10,11]. When the maximum tolerated dose is administrated, levels of HbF may range from 10 to 40%, which significantly improves the clinical manifestations of the disease, such as vaso-occlusive crisis (VOCs) and painful crisis, bone necrosis, fewer episodes of acute chest syndrome, hospitalizations and blood transfusions, consequently reducing the mortality rate [9,12].

But even the response to treatment with HU is highly variable and seems to be an inherited trait [8,13]. About 25% of patients do not respond to HU treatment or are considered poor metabolizers [8]. The presence of variants in genes responsible not only for HU metabolism but also for regulating HbF expression and erythroid progenitor proliferation might be the answer for the differences in patient responses to HU [14]. Identifying those variants that might predict the response to HU is important to, in the future, in situations where therapeutic alternatives are a possibility, prevent HU being prescribed to those who will not respond to treatment, saving time and means, as well as not exposing individuals to a drug that might cause side effects, as HU is known to cause gastrointestinal toxicity, such as nausea and anorexia, cytopenia, hyperpigmentation, weight gain and possible teratogenic effects [7,13]. Even though HU impacts the metabolome of treated patients and the efficacy and toxicity in long term treatments, especially in children, which is not fully understood [11], HU is considered safe and effective in adults and children with SCA [6,9].

In the present pharmacogenetic study, we intended to identify variants associated with drug response that might allow in the future to distinguish responders from non-responders to HU and even individuals that might develop adverse effects to this specific drug. In that sense, we analyzed the exons of 77 genes described in the literature as potentially associated with HU metabolism in Angolan children treated with HU and followed up at Hospital Pediátrico David Bernardino and Hospital Geral do Bengo. Evaluation of drug response took into consideration HbF levels, other hematological and biochemical parameters, hemolysis, and number of VOCs and hospitalizations after 6 and 12 months of treatment.

## 2. Results

### 2.1. Clinical Findings

The study sample included 191 SCA Angolan children of both genders (52.4% females), aged 3 to 12 years (mean ± SD: 6.53 ± 2.58). Of the 191 children, 40 dropped out of the study before treatment. The remaining 151 were eligible and started the treatment with HU, but only 138 regularly attended the physicians’ appointments and completed at least 12 months of treatment (54.3% females, mean age at the beginning of the treatment ± SD: 7.71 ± 2.68).

Follow-up of the eligible patients who started treatment with HU included periodic hematological and biochemical analysis to ensure no toxic events were associated with the medicine. After 12 months of treatment, significant increases were observed in HbF, total hemoglobin, mean corpuscular volume (MCV), mean corpuscular hemoglobin (MCH) and alanine aminotransferase (ALT), as well as significant decreases in erythrocytes (RBC), white blood cells (WBC), reticulocytes, platelets, and total bilirubin (*p*-value < 0.05). The number of transfusions and hospitalizations also decreased significantly (Table 1).

Of the 138 participants who completed at least 12 months of HU treatment, 81 were considered responders (61.8%), 50 non-responders/poor responders (38.2%), and 7 non-compliant to treatment. No significant differences were identified between drug response and gender or age. The hematological, biochemical and clinical pre- and post-treatment values in responders and non-/poor responders are presented in Table 1.

### 2.2. Genetic Findings

In the 77 genes sequenced, 1299 different variants were identified, being 1279 single-nucleotide variants (SNVs), 15 deletions and 5 insertions (Supplementary Table S1). In absolute numbers, 30,850 variants were identified (30,606 SNVs, 208 deletions and 36 insertions). The most prevalent were missense variants (47.27%), followed by synonymous variants (37.95%).

**Figure 1:**
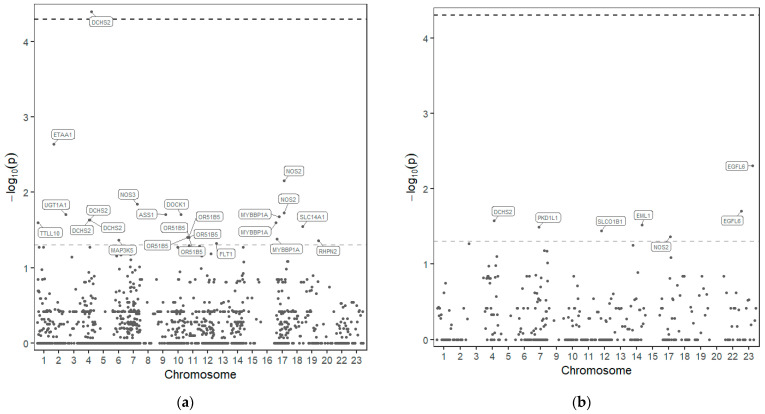
Manhattan plot—distribution of *p*-values of the association of SNP in the 77 genes sequenced and response to HU. SNPs above the upper line are considered significant after *p*-value correction. (**a**) Dominant model; (**b**) Recessive model.

From the 1279 variants identified, 30 variants in 18 of those genes seem to be associated with drug response to HU (Figure 1 and Table 2).

## 3. Discussion

The present results show that the use of HU in children with SCA should be considered safe and beneficial. Even in children considered non-/poor responders, using HbF as the main criteria, significant changes were registered in almost the same parameters identified in responders, which also denotes clinical improvement in these patients. 

The percentage of non-/poor responders was higher than expected. Possibly the fixed dose of 20 mg/kg was not enough for all the children, and it would have been more effective for some children to use a higher or the maximum tolerated dose.

Between the groups (responders vs. non-/poor responders), pre-treatment values had no significant differences except for HbF and total hemoglobin (*p*-value = 0.005 and *p*-value = 0.02). These differences are probably due to the presence of variants that affect HbF expression per se and may also affect or reverberate the drug action.

In 18 genes out of the 77 sequenced, 30 variants could be associated with HU response, although only one was considered significant after *p*-value correction, rs10017772 in the DCHS2 gene (dachsous cadherin-related 2) (*p*-value before correction <0.001 and after correction 0.045).

**Figure 2:**
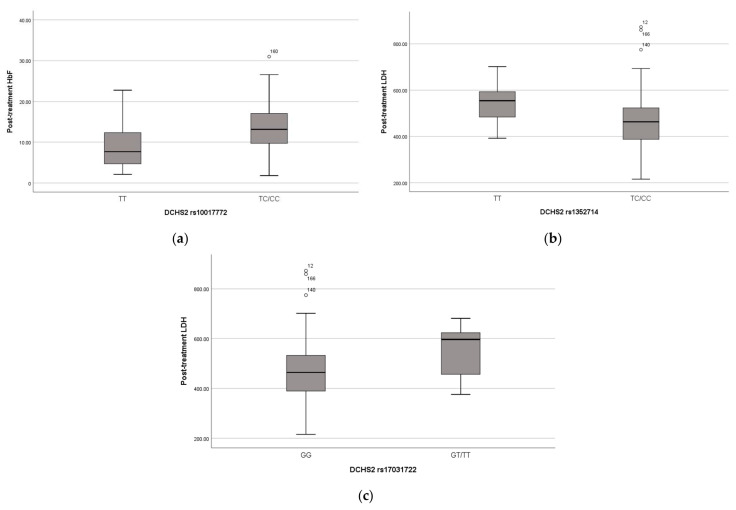
(**a**) Distribution of HbF levels (%) after treatment and DCHS2 SNP rs10017772; (**b**) Distribution of LDH levels (U/L) after treatment and DCHS2 SNP rs1352714; (**c**) Distribution of LDH levels (U/L) after treatment and DCHS2 SNP rs1703122. Lines of the boxes represent the lower quartile, median, and upper quartile, and the outliers are the points outside the whiskers.

The missense variant in the DCHS2 gene was found in 101 children in heterozygosity and in 24 children in homozygosity. The presence of this variant seems to have a positive effect on these children’s phenotypes, as the heterozygous and homozygous carriers have significantly higher HbF levels pre- (*p*-value = 0.008) (Figure 2a) and post-treatment (*p*-value < 0.001) and lower WBC (*p*-value = 0.023), neutrophils (*p*-value = 0.027) and platelet (*p*-value = 0.013) counts post-treatment.

The other 4 variants in the DCHS2 gene were identified as possibly associated with the drug response to HU. The presence of 3 variants in linkage, rs12500437, rs13109747 and rs140019361, identified in 92 children in heterozygosity and 31 children in homozygosity in the DCHS2 gene, even in heterozygosity, seems to be an advantage for response to HU treatment. In the heterozygous for these variants, significant differences were also observed on the total number of hospitalizations (*p*-value = 0.016), LDH levels (*p*-value = 0.003) and the number of pain crises (*p*-value = 0.02) after treatment. On the other hand, the presence of the missense variant rs79295524 in homozygosity (identified in 69 heterozygous and 14 homozygous children) correlates with a poorer response to HU treatment.

Three other variants in the DCHS2 gene were associated with typical clinical manifestations of SCD and hemolysis biomarkers, as variant rs17373874 (identified in 96 heterozygous and 52 homozygous children, *p*-value = 0.01, recessive model) is associated with pain crises, variant rs1352714 (identified in 85 heterozygous and 83 homozygous children, *p*-value = 0.005, dominant model) with lower LDH post-treatment and rs17031722 (identified in 12 heterozygous and 2 homozygous children, *p*-value = 0.017, dominant model) with higher LDH post-treatment (Figure 2b,c).

The DCHS2 gene is located on chromosome 4q31.3 and was first identified by bioinformatic analysis [15] with several cadherin motifs distributed over the exons. With 24 exons, it encodes a large protein, protocadherin 23, with 3371 amino acids, classified as a member of the cadherin family, which includes calcium-dependent cell-adhesion proteins. A polymorphism in this gene (rs61746132) had already been identified by Sheehan et al. as correlated with changes in HbF (%) from baseline to final (ΔHbF) (*p*-value 1.73 × 10^−4^) [16]. In our studied population, this variant was identified in 61 heterozygous and 9 homozygous children, and the association with drug response was not significant, although we observed a tendency of higher ΔHbF in the presence of the A allele and a correlation with strokes (*p*-value = 0.02).

We identified a synonymous variant in the TTLL10 gene associated with drug response in 43 heterozygous (rs57556493) and with HbF levels after treatment (*p*-value = 0.017). This gene encodes for TTLL10 protein (tubulin tyrosine ligase like 10), a glycylase for nucleosome assembly protein 1 (NAP1) and with tubulin glycase activity [17]. Associations in this gene were also detected by other studies [16].

In the PKD1L1 gene, we found a synonymous variant, present in 86 heterozygous carriers and 34 homozygous, that in the recessive model is considered to be associated with poorer response to HU. Gene PKD1L1 encodes for a polycystin-1-like protein, associated with the left-right asymmetry, being pathogenic homozygous polymorphisms in this gene related with laterality de-fects in humans [18].

In gene RHPN2, a missense variant (rs28626308) considered probably damaging, was identified in 27 heterozygous and 1 homozygous child, and it was associated with poorer response. This validates previous findings by Sheehan et al., where this variant was considered significantly associated with variation in HbF after HU treatment [16]. We also found a significant association with strokes (*p*-value = 0.012 dominant model). RHPN2 encodes for a protein called RhoA-binding protein, Rhopilin-2, that regulates the organization of actin cytoskeleton [19].

The EML1 gene encodes for the protein Echinoderm microtubule-associated protein-like 1, and one missense variant has already been identified as associated with variations in HbF after HU treatment (rs141631682) [16]. This variant was present in 6 heterozygous children, but we found no association with any of the clinical or laboratory data. In our population, another missense variant was identified as potentially associated with HU response (rs34198557). This variant was found in 50 heterozygous and 8 homozygous. Its presence in homozygosity is associated with lower HbF after treatment (*p*-value = 0.006) and poorer response to treatment.

MYBBP1A is a gene that encodes for a transcriptional regulator, with a major role in the cell cycle, mitosis, cellular senescence, and even epigenetic regulation [20]. In our study, a missense variant (rs899441) considered possibly damaging by PolyPhen showed significant association with a response to HU, variation in HbF (*p*-value = 0.031) and total hemoglobin after treatment (*p*-value = 0.036) in the dominant model. This variant had already been associated with HbF levels after treatment [16]. Two other missense variants (rs73972683 and rs78578064) were found to be associated with drug response and with pain crises per year after HU (*p*-value = 0.026 and *p*-value = 0.031).

Two intronic variants and one short tandem repeat in the promotor of gene MAP3K5 have been previously described as influencers in HU response [14,21,22]. The synonymous variant, rs9389412, was found in 15 heterozygous carriers, and the presence of the variant allele seems to be a disadvantage as the RBC after treatment is significantly lower in these children (*p*-value = 0.013), and WBC and neutrophil count are significantly higher (*p*-values < 0.001 and *p*-value = 0.012 respectively).

The UGT1A1 gene encodes for the enzyme UDP glucuronyl transferase 1 responsible for bilirubin conjugation. Alterations in the TATA box of the gene promoter region have been studied on SCA, where the presence of seven or more TA repeats (instead of the six in wild type) are associated with increased bilirubin levels and cholelithiasis; and even with HU treatment, bilirubin levels do not go to normal levels [23,24,25]. In this SCA population, only one synonymous variant was associated with response to HU treatment (rs28900406). This variant was identified in four heterozygous patients, all non-/poor responders.

A previous genome-wide association study suggested that polymorphisms in the 5′ olfactory receptor gene cluster could be associated with HbF levels, as they are located upstream of the HBB cluster locus and might regulate the expression of the HBG2 gene [26,27]. A frameshift variant in gene OR51B5 (rs147062602), although not significantly associated with HU response, is in fact associated with HbF and total hemoglobin values before (*p*-value = 0.002 and *p*-value = 0.017) and after treatment (*p*-value = 0.019 and *p*-value = 0.037). It is also associated with the number of transfusions (*p*-value = 0.003) and hospitalizations (*p*-value = 0.019) per year without HU treatment. Moreover, five variants in linkage (rs12273630, rs57273781, rs57900141, rs58233587 and rs61738485) were associated with HbF before treatment (*p*-value = 0.011), post-treatment (*p*-value = 0.020) and transfusions (*p*-value = 0.08).

In gene FLT1, one synonymous variant (rs7993418) stood out as possibly associated with the drug response to HU. This variant was found in 72 heterozygous and 21 homozygous children, and its presence is associated with higher changes in HbF levels after treatment. The FLT1 gene, also known as VEGFR1, encodes for a tyrosine kinase receptor, a member of the vascular endothelial growth factor receptor family, with an important role in cell proliferation and differentiation. Ma et al. had already identified in 2007 that five SNPs in this gene were associated with significant changes in HbF (% and g/dL) [14]. Previous studies have already found significant associations with this specific variant (rs7993418) and the response to ranibizumab [28,29]. 

Located in the X chromosome, the EGFL6 gene encodes for a family member of epidermal growth factors [30]. In the recessive model, we found two missense variants where the presence of the minor allele (A) is associated with poorer response to HU, rs16979010 and rs16979033, being the last one associated with HbF and total hemoglobin values after HU treatment (*p*-value = 0.032 and *p*-value = 0.028). Ma et al. also established associations between the gene EGFL6 and HbF levels [14].

The synonymous variant rs2291075 in Gene SLCO1B1, which has been previously associated with the pharmacokinetics of tacrolimus and some chemotherapeutic drugs [31,32], and which was present in 91 heterozygous and 66 homozygous children, was associated with response to HU treatment through the recessive model, its presence being an advantage. Gene SLCO1B1 encodes for an organic anion transporting polypeptide, the OATP1B1, HU being one substrate of this transporter.

Gene SLC14A1 encodes for a urea transporter (UTB), highly expressed in erythrocytes membranes, that allows HU diffusion bidirectionally. We identified a missense variant, rs1058396, considered benign by PolyPhen, that is probably associated with a poorer response to HU treatment in a dominant model. Previous studies identified polymorphisms in this gene to be associated with HU pharmacokinetics and efficacy [12,33,34].

Four other variants in genes NOS2, NOS3 and ASS1 were identified that seem to be associated with drug response. For the variant rs3918211, found in 63 heterozygous and 8 homozygous children, significant differences were identified in HbF levels after treatment (*p*-value = 0.030, dominant model). Variant rs16966563 in NOS2 was found in 80 heterozygous and 9 homozygous children and seems to be associated with laboratory data after treatment: total hemoglobin (*p*-value = 0.008), RBC (*p*-value = 0.009), WBC (*p*-value = 0.014), neutrophils (*p*-value = 0.014), LDH (*p*-value = 0.018) and number of transfusions after treatment (*p*-value = 0.027), the presence of the variant allele being an advantage. Variant rs3730017 in NOS2 was found in 80 heterozygous and 9 homozygous children and seems to be associated with laboratory data after treatment: total hemoglobin (*p*-value = 0.008), RBC (*p*-value = 0.011), WBC (*p*-value = 0.047), LDH (*p*-value = 0.020) and number of transfusions after treatment (*p*-value = 0.027), the presence of the alternative allele being an advantage. Other studies have also shown associations between polymorphisms in genes ASS1, NOS2A and ARG2 with HU treatment efficacy in SCD/B-thalassemia patients [22,35]. Future use of these genes in clinical routine could be interesting; however, more studies are needed in order to confirm the association.

In the present study, we verified that several polymorphisms seem to be associated with response to HU treatment in SCA patients, as previously reported in other studies. The limitations of this study include the use of a fixed dose, a restricted population of Angolan children and the unexpected high drop-off rate. Therefore, studies with higher sample sizes that compare fixed dose with maximum tolerated dose are needed to confirm these associations and to achieve the main objective of distinguishing previous individuals that might benefit from the use of HU treatment (responders) from the ones who will not (non-responders) or that might develop severe adverse effects, moving towards personalized medicine in SCA.

## 4. Materials and Methods

### 4.1. Patient Selection

The study sample consisted of 191 SCA Angolan children (homozygous for the β^s^ allele), followed up at Hospital Pediátrico David Bernardino and Hospital Geral do Bengo. To be included in this study, patients should not have been treated with HU or had a blood transfusion in the previous three months.

In the first consultation, a full anamnesis was performed, which included previous manifestations of the disease and current symptoms. A neurological and physical exam was also conducted.

### 4.2. Hematological and Biochemical Analysis

In the first consultation, each patient was subjected to clinical examination by a specialized pediatrician and collection of a whole blood sample, used for hematological and biochemical analysis, electrophoresis for SCA confirmation and HbF quantification.

Complete blood count (RBC, reticulocytes, WBC and platelets), hemoglobin, MCV, and MCH were determined using the XT-2000i Hematology Analyzer (Sysmex Corporation, Kobe, Japan). The hemoglobin fractions, including HbF, were quantified by HPLC (Biorad Variant II, Hercules, CA, USA).

Biochemical blood tests included LDH, creatinine, urea, total and direct bilirubin, aspartate aminotransferase (AST) and alanine aminotransferase (ALT) levels, which were determined using a Cobas C111 (Roche Diagnostics, Basel, Switzerland) and a Mindray BA-88A (Mindray, Shenzhen, China).

The eligible children started the treatment with HU (daily dose 20 mg/kg), and every month had a follow-up consultation, where all the manifestations and symptoms were registered. Hematological and biochemical analysis, as well as HbF determination, were repeated every three months.

### 4.3. Sequencing Analysis

Genomic DNA was extracted and purified from peripheral blood samples using the QIAamp DNA Blood Mini Kit (Qiagen GmbH, Hilden, Germany) according to the manufacturer’s recommendations. All samples were quantified by a Qubit™ dsDNA HS fluorometric assay (ThermoFisher Scientific Inc., Waltham, MA, USA).

A custom enrichment panel was used for sequencing the exons of 77 genes associated with HU metabolism (Supplementary Table S2). The libraries were prepared with KAPA HyperCap and KAPA UDI Primers (Roche Sequencing Solutions, Pleasanton, CA, USA), and their quality was assessed with High Sensitivity D1000 reagents on an Agilent 4200 TapeStation System. Paired-end sequencing was performed on a NextSeq 550 sequencer (Illumina, San Diego, CA, USA) using the NextSeq 500/550 Mid-Output kit v2 (300 cycles). Samples were aligned with the reference GRCh37/hg19 human genome and variant analysis was performed using the Illumina Variant Studio V.3.0. 

### 4.4. Response to Treatment Classification

The response to HU treatment was evaluated based on the changes in HbF, and participants were classified as responders, non-/poor responders or non-compliant. Responders had to achieve HbF above 20% or an increase of at least 5% from the baseline [36] after 12 months of treatment. Compliance was evaluated by changes in hematological and biochemical parameters (increase in total hemoglobin, MCV, MCH, and decrease in reticulocyte count, WBC, neutrophils, platelets and bilirubin) [36] to exclude non-compliant participants from the non-/poor responders group.

### 4.5. Statistical Analysis

To compare hematological and biochemical data, as well as phenotypic characteristics, paired sample *t*-tests, independent sample t-tests and qui-square tests or the alternative Fisher’s exact tests were used whenever conditions were appropriate.

An algorithm was developed for the data collected from Illumina’s variant studio with the purpose of detecting any variability in each allele coordinate. Association between polymorphisms and drug response was determined using a Fisher’s exact test per unique coordinate within each gene. The Benjamini and Hochberg false discovery rate (FDR) procedure was applied to correct multiple testing. After multiple testing correction, all *p*-values under 0.05 were considered significant. This approach was applied considering two genetic models: dominant and recessive.

All statistical analysis was performed using the IBM SPSS software version 26.0 (IBM Corp, Armonk, NY, USA) and R version 4.2.2.

## Figures and Tables

**Table 1:** Pre- and post-treatment clinical, hematological and biochemical data of the 138 patients on HU treatment and in responders and non-/poor responders to HU treatment (mean ± SD; *p*-value paired samples *t*-test) and on responders and non-responders.

	Patients on HU for 12 Months (*n* = 138)			Non-/Poor Responders (*n* = 50)			Responders (*n* = 81)		
	Pre-Treatment	Post-Treatment	*p*-Value	Pre-Treatment	Post-Treatment	*p*-Value	Pre-Treatment	Post-Treatment	*p*-Value
**Fetal hemoglobin (%)**	5.96 ± 3.93	12.22 ± 6.03	**<0.001**	4.68 ± 3.87	7.26 ± 3.65	**<0.001**	6.63 ± 3.81	15.57 ± 4.92	**<0.001**
**Total hemoglobin (g/dL)**	7.46 ± 0.90	7.99 ± 0.97	**<0.001**	7.13 ± 0.97	7.53 ± 0.92	**<0.001**	7.61 ± 0.76	8.27 ± 0.89	**<0.001**
**RBC (10^12^/L)**	2.99 ± 0.60	2.83 ± 0.52	**<0.001**	2.87 ± 0.62	2.73 ± 0.52	**0.005**	3.04 ± 0.54	2.88 ± 0.52	**<0.001**
**MCV (fL)**	76.86 ± 7.69	85.07 ± 9.01	**<0.001**	76.93 ± 7.23	83.67 ± 7.57	**<0.001**	76.77 ± 7.85	86.29 ± 9.47	**<0.001**
**MCH (pg)**	25.42 ± 2.91	28.88 ± 3.50	**<0.001**	25.31 ± 2.84	28.16 ± 2.96	**<0.001**	25.43 ± 2.90	29.48 ± 3.62	**<0.001**
**WBC (10^9^/L)**	13.46 ± 3.81	9.87 ± 2.36	**<0.001**	13.39 ± 4.11	10.45 ± 2.21	**<0.001**	13.70 ± 3.62	9.49 ± 2.44	**<0.001**
**Neutrophils (10^9^/L)**	5.70 ± 1.90	4.18 ± 1.21	**<0.001**	5.73 ± 1.94	4.53 ± 1.15	**<0.001**	6.74 ± 1.89	3.95 ± 1.24	**<0.001**
**Platelets (10^9^/L)**	447.63 ± 173.51	404.96 ± 121.77	**<0.001**	433.06 ± 164.36	416.99 ± 111.98	0.37	456.98 ± 182.8	393.47 ± 123.14	**<0.001**
**Reticulocytes (%)**	9.87 ± 3.75	6.85 ± 2.82	**<0.001**	10.07 ± 3.44	7.67 ± 3.01	**<0.001**	9.80 ± 3.94	6.33 ± 2.65	**<0.001**
**Creatinine (mg/dL)**	0.51 ± 0.60	0.43 ± 0.18	0.109	0.54 ± 0.64	0.42 ± 0.20	0.184	0.50 ± 0.60	0.44 ± 0.17	0.299
**LDH (U/L)**	484.82 ± 184.32	476.32 ± 116.41	0.597	520.83 ± 202.73	491.30 ± 120.02	0.313	458.18 ± 172.84	467.91 ± 116.85	0.632
**Urea (mg/dL)**	30.79 ± 18.55	33.19 ± 9.58	0.146	34.90 ± 18.12	34.65 ± 10.74	0.978	28.08 ± 18.31	31.96 ± 7.63	0.062
**Direct Bilirubin (mg/dL)**	0.55 ± 0.91	0.57 ± 0.27	0.821	0.75 ± 1.29	0.50 ± 0.18	0.176	0.45 ± 0.59	0.58 ± 0.28	**0.048**
**Total Bilirubin (mg/dL)**	1.38 ± 1.44	1.05 ± 0.51	**0.008**	1.35 ± 1.44	1.17 ± 0.58	0.374	1.36 ± 1.44	0.92 ± 0.37	**0.01**
**AST (U/L)**	34.12 ± 16.41	35.92 ± 22.99	0.447	34.41 ± 16.20	34.64 ± 11.15	0.925	33.31 ± 16.81	36.63 ± 28.65	0.368
**ALT (U/L)**	11.08 ± 9.72	18.10 ± 28.62	**0.006**	11.15 ± 8.52	15.07 ± 5.83	**<0.001**	11.00 ± 10.54	20.11 ± 37.03	**0.036**
**Transfusions/year**	0.39 ± 0.53	0.14 ± 0.57	**<0.001**	0.53 ± 0.74	0.24 ± 0.85	**0.044**	0.30 ± 0.33	0.04 ± 0.19	**<0.001**
**Hospitalizations/year**	0.47 ± 0.48	0.16 ± 0.47	**<0.001**	0.57 ± 0.57	0.16 ± 0.42	**<0.001**	0.42 ± 0.41	0.10 ± 0.34	**<0.001**

RBC—erythrocytes; MCV—mean corpuscular volume; MCH—mean corpuscular hemoglobin; WBC—white blood cells; LDH—lactate dehydrogenase; AST—aspartate aminotransferase; ALT—alanine aminotransferase.

**Table 2:** Variants associated with HU response.

Chr	Gene	SNP	Variant	Consequence	*p*-Value *	*p*-Value **	Model
1	TTLL10	rs57556493	C > T	synonymous variant	0.025	1	dominant
2	ETAA1	rs10209114	C > T	synonymous variant	0.002	1	dominant
2	UGT1A1	rs28900406	C > T	synonymous variant	0.020	1	dominant
4	DCHS2	rs10017772	T > C	missense variant	<0.001	0.045	dominant
4	DCHS2	rs12500437	G > T	missense variant	0.023	1	dominant
4	DCHS2	rs13109747	C > T	synonymous variant	0.023	1	dominant
4	DCHS2	rs140019361	TTTTG > T	frameshift variant	0.023	1	dominant
4	DCHS2	rs79295524	G > C	missense variant	0.027	1	recessive
6	MAP3K5	rs9389412	T > C	synonymous variant	0.043	1	dominant
7	NOS3	rs3918211	T > C	synonymous variant	0.014	1	dominant
7	PKD1L1	rs885337	A > G	synonymous variant	0.032	1	recessive
9	ASS1	rs62637575	A > G	missense variant	0.020	1	dominant
10	DOCK1	rs2229599	C > T	synonymous variant	0.020	1	dominant
11	OR51B5	rs12273630	C > T	missense variant	0.040	1	dominant
11	OR51B5	rs57273781	G > T	missense variant	0.040	1	dominant
11	OR51B5	rs57900141	T > C	missense variant	0.040	1	dominant
11	OR51B5	rs58233587	G > A	synonymous variant	0.040	1	dominant
11	OR51B5	rs61738485	C > T	synonymous variant	0.040	1	dominant
12	SLCO1B1	rs2291075	C > T	synonymous variant	0.036	1	recessive
13	FLT1	rs7993418	G > G/A	synonymous variant	0.048	1	dominant
14	EML1	rs34198557	C > T	missense variant	0.030	1	recessive
17	MYBBP1A	rs73972683	C > T	missense variant	0.042	1	dominant
17	MYBBP1A	rs78578064	T > C	missense variant	0.025	1	dominant
17	MYBBP1A	rs899441	T > C	missense variant	0.021	1	dominant
17	NOS2	rs16966563	T > C	synonymous variant	0.007	1	dominant
17	NOS2	rs3730017	G > A	missense variant	0.019	1	dominant
18	SLC14A1	rs1058396	G > A	missense variant	0.028	1	dominant
19	RHPN2	rs28626308	C > T	missense variant	0.044	1	dominant
X	EGFL6	rs16979010	G > A	missense variant	0.020	1	recessive
X	EGFL6	rs16979033	G > A	missense variant	0.005	1	recessive

* *p*-value, ** *p*-value after correction, Chr—chromosome.

## Data Availability

The data that support the findings of this study are available from the corresponding author (MB) upon reasonable request.

## Supplementary Materials

The following supporting information can be downloaded at https://www.mdpi.com/article/10.3390/ijms24108792/s1.
