# Supplementary material for: Are Genetic Modifiers the Answer to Different Responses to Hydroxyurea Treatment?—A Pharmacogenetic Study in Sickle Cell Anemia Angolan Children
Source: Int J Mol Sci. 2023 May 15;24(10):8792. doi: 10.3390/ijms24108792 (PMC10218819; doi:10.3390/ijms24108792)
Supplement: Supplementary file 1 [file ijms-24-08792-s001.zip › ijms-2316219-supplementary/Supplementary Table S1.pdf]

Table S1. Identified variants (Reference genome h38).

Chr	Gene	Variant	Coordinate	Type
1	ADAR	C>T	154560725	snv
1	ADAR	C>T	154561925	snv
1	ADAR	C>T	154569626	snv
1	ADAR	T>C	154570903	snv
1	ADAR	T>C	154573913	snv
1	ADAR	T>C	154573967	snv
1	ADAR	T>A	154574115	snv
1	ADAR	G>A	154574227	snv
1	ADAR	C>G	154574462	snv
1	ADAR	T>C	154574820	snv
1	ADAR	G>A	154574891	snv
1	ADAR	G>A	154574933	snv
1	ADAR	C>T	154575040	snv
1	ADCY10	G>A	167778933	snv
1	ADCY10	A>G	167778996	snv
1	ADCY10	A>G	167779982	snv
1	ADCY10	A>G	167780083	snv
1	ADCY10	T>A	167787403	snv
1	ADCY10	G>A	167787415	snv
1	ADCY10	T>C	167791328	snv
1	ADCY10	G>A	167792339	snv
1	ADCY10	A>G	167792341	snv
1	ADCY10	C>A	167792342	snv
1	ADCY10	G>A	167793767	snv
1	ADCY10	A>G	167798535	snv
1	ADCY10	G>A	167798559	snv
1	ADCY10	T>C	167802276	snv
1	ADCY10	A>G	167802389	snv
1	ADCY10	G>A	167805777	snv
1	ADCY10	C>T	167814905	snv
1	ADCY10	A>G	167815030	snv
1	ADCY10	A>G	167817639	snv
1	ADCY10	G>A	167823663	snv
1	ADCY10	T>C	167825485	snv
1	ADCY10	A>G	167825606	snv
1	ADCY10	A>G	167830227	snv
1	ADCY10	T>C	167830254	snv
1	ADCY10	G>A	167839578	snv
1	ADCY10	C>T	167847696	snv
1	ADCY10	A>G	167849414	snv
1	ADCY10	G>A	167865871	snv
1	ADCY10	C>T	167870911	snv

1	ADCY10	C>T	167871012	snv
1	HDAC1	AAGG>A	32798325	deletion
1	HAO2	T>C	119923317	snv
1	HAO2	T>C	119923724	snv
1	HAO2	G>A	119923751	snv
1	HAO2	C>A	119927408	snv
1	HAO2	C>T	119927484	snv
1	HAO2	T>C	119929250	snv
1	HAO2	C>A	119934879	snv
1	HDAC1	G>A	32782358	snv
1	HDAC1	C>T	32794734	snv
1	HDAC1	A>G	32797082	snv
1	HDAC1	G>A	32797160	snv
1	TAL1	CCCT>C	47685639	deletion
1	TTLL10	AC>A	1115550	deletion
1	TTLL10	CG>C	1116222	deletion
1	TAL1	T>C	47685455	snv
1	TAL1	G>A	47685569	snv
1	TAL1	G>C	47691120	snv
1	TAL1	G>A	47691250	snv
1	TAL1	C>A	47691251	snv
1	TAL1	C>T	47691350	snv
1	TAL1	G>C	47691481	snv
1	TTLL10	C>T	1114650	snv
1	TTLL10	G>A	1114668	snv
1	TTLL10	G>A	1114699	snv
1	TTLL10	G>A	1115071	snv
1	TTLL10	C>T	1115415	snv
1	TTLL10	G>A	1115425	snv
1	TTLL10	G>A	1115454	snv
1	TTLL10	C>T	1115461	snv
1	TTLL10	C>T	1115510	snv
1	TTLL10	C>A	1115604	snv
1	TTLL10	C>T	1116182	snv
1	TTLL10	G>A	1116189	snv
1	TTLL10	G>A	1116227	snv
1	TTLL10	T>C	1116231	snv
1	TTLL10	C>T	1118275	snv
1	TTLL10	C>T	1118357	snv
1	TTLL10	G>A	1119343	snv
1	TTLL10	A>G	1119361	snv
1	TTLL10	C>T	1119399	snv

1	TTLL10	G>A	1119430	snv
1	TTLL10	C>G	1120370	snv
1	TTLL10	G>A	1120431	snv
1	TTLL10	A>C	1120488	snv
1	TTLL10	G>A	1120494	snv
1	TTLL10	T>C	1120495	snv
1	TTLL10	G>A	1120503	snv
1	TTLL10	T>C	1132870	snv
1	TTLL10	G>C	1132937	snv
1	TTLL10	C>T	1132952	snv
1	TTLL10	C>A	1132966	snv
1	TTLL10	C>T	1132976	snv
1	TTLL10	C>T	1133033	snv
1	TTLL10	G>A	1133073	snv
1	TTLL10	A>G	1133077	snv
1	TTLL10	G>A	1133125	snv
2	ETAA1	C>T	67624610	snv
2	ETAA1	G>T	67624628	snv
2	ETAA1	C>T	67624779	snv
2	ETAA1	C>T	67626338	snv
2	ETAA1	A>G	67630027	snv
2	ETAA1	C>G	67630472	snv
2	ETAA1	T>C	67630476	snv
2	ETAA1	T>C	67630542	snv
2	ETAA1	C>G	67630835	snv
2	ETAA1	G>A	67630980	snv
2	ETAA1	G>A	67631064	snv
2	ETAA1	G>A	67631395	snv
2	ETAA1	T>C	67631541	snv
2	ETAA1	A>G	67631578	snv
2	ETAA1	C>T	67631611	snv
2	ETAA1	A>G	67631800	snv
2	ETAA1	G>A	67631831	snv
2	ETAA1	A>G	67631868	snv
2	ETAA1	A>G	67632016	snv
2	ETAA1	C>T	67632125	snv
2	ETAA1	C>A	67632348	snv
2	ETAA1	A>G	67632397	snv
2	ETAA1	A>G	67632424	snv
2	ETAA1	T>C	67632429	snv
2	ETAA1	G>A	67637150	snv
2	FZD5	G>A	208631748	snv
2	FZD5	G>A	208631784	snv
2	FZD5	G>A	208631933	snv
2	FZD5	G>A	208631983	snv

2	FZD5	A>G	208632528	snv
2	FZD5	A>G	208632780	snv
2	FZD5	C>T	208633009	snv
2	FZD5	G>A	208633374	snv
2	FZD5	C>T	208633377	snv
2	FZD5	T>C	208633413	snv
2	UGT1A1	G>A	234669092	snv
2	UGT1A1	T>C	234669259	snv
2	UGT1A1	C>T	234676942	snv
2	UGT1A1	C>T	234681031	snv
3	CCR5	G>A	46414529	snv
3	CCR5	T>C	46414618	snv
3	CCR5	C>T	46414712	snv
3	CCR5	C>T	46415066	snv
3	CCR5	G>A	46415394	snv
3	CCR5	C>T	46415397	snv
3	CCR5	A>T	46415409	snv
3	CCR5	TACAGTCAGTATCAATTCTGGAAGAATTTCCAG>T	46414943	deletion
3	GATA2	C>T	128204951	snv
3	GATA2	G>A	128205108	snv
3	GATA2	G>C	128205701	snv
3	GATA2	G>C	128205809	snv
3	GATA2	G>C	128205860	snv
3	PHC3	T>C	169834951	snv
3	PHC3	G>A	169834952	snv
3	PHC3	C>T	169840471	snv
3	PHC3	T>C	169846729	snv
3	PHC3	T>C	169846767	snv
3	PHC3	C>G	169846888	snv
3	PHC3	A>G	169846994	snv
3	PHC3	C>T	169847125	snv
3	PHC3	T>A	169889209	snv
3	PHC3	T>C	169889211	snv
3	PHC3	C>T	169896684	snv
4	DCHS2	TTTTG>T	155244401	deletion
4	DCHS2	G>A	155155914	snv
4	DCHS2	G>C	155155969	snv
4	DCHS2	G>A	155156001	snv
4	DCHS2	C>T	155156138	snv
4	DCHS2	G>A	155156207	snv
4	DCHS2	G>A	155156247	snv
4	DCHS2	G>A	155156412	snv
4	DCHS2	G>A	155156542	snv

4	DCHS2	G>A	155156598	snv
4	DCHS2	G>A	155156674	snv
4	DCHS2	T>A	155156750	snv
4	DCHS2	T>G	155156875	snv
4	DCHS2	T>C	155157015	snv
4	DCHS2	C>T	155157404	snv
4	DCHS2	C>T	155157531	snv
4	DCHS2	C>T	155157646	snv
4	DCHS2	C>T	155157672	snv
4	DCHS2	C>T	155157729	snv
4	DCHS2	G>A	155157730	snv
4	DCHS2	A>G	155157871	snv
4	DCHS2	A>G	155157953	snv
4	DCHS2	C>T	155157961	snv
4	DCHS2	C>T	155158081	snv
4	DCHS2	A>C	155158090	snv
4	DCHS2	G>A	155158104	snv
4	DCHS2	A>G	155160429	snv
4	DCHS2	T>C	155161713	snv
4	DCHS2	A>G	155161887	snv
4	DCHS2	G>C	155163829	snv
4	DCHS2	A>T	155163847	snv
4	DCHS2	C>T	155176739	snv
4	DCHS2	T>A	155176771	snv
4	DCHS2	T>C	155180721	snv
4	DCHS2	C>G	155219318	snv
4	DCHS2	A>G	155219338	snv
4	DCHS2	A>G	155219361	snv
4	DCHS2	A>G	155219373	snv
4	DCHS2	T>C	155219513	snv
4	DCHS2	G>C	155219662	snv
4	DCHS2	C>A	155219788	snv
4	DCHS2	A>G	155225932	snv
4	DCHS2	G>A	155225951	snv
4	DCHS2	G>T	155225953	snv
4	DCHS2	C>T	155226021	snv
4	DCHS2	G>A	155226252	snv
4	DCHS2	G>A	155241572	snv
4	DCHS2	C>G	155241663	snv
4	DCHS2	G>A	155241674	snv
4	DCHS2	G>A	155241937	snv
4	DCHS2	G>A	155242011	snv
4	DCHS2	C>A	155243523	snv
4	DCHS2	C>T	155243573	snv
4	DCHS2	G>A	155243574	snv

4	DCHS2	A>G	155243603	snv
4	DCHS2	T>C	155243604	snv
4	DCHS2	G>A	155243612	snv
4	DCHS2	T>G	155244464	snv
4	DCHS2	G>T	155244475	snv
4	DCHS2	G>A	155249289	snv
4	DCHS2	G>C	155250701	snv
4	DCHS2	C>T	155250749	snv
4	DCHS2	G>A	155253684	snv
4	DCHS2	C>T	155253808	snv
4	DCHS2	C>G	155253919	snv
4	DCHS2	C>T	155253964	snv
4	DCHS2	C>T	155254051	snv
4	DCHS2	G>C	155254071	snv
4	DCHS2	C>T	155254428	snv
4	DCHS2	C>A	155254512	snv
4	DCHS2	A>G	155254523	snv
4	DCHS2	G>A	155254558	snv
4	DCHS2	C>G	155256080	snv
4	DCHS2	A>G	155256177	snv
4	DCHS2	G>A	155256205	snv
4	DCHS2	T>C	155287377	snv
4	DCHS2	G>T	155287482	snv
4	DCHS2	T>C	155287535	snv
4	DCHS2	A>G	155298424	snv
4	DCHS2	G>T	155410458	snv
4	DCHS2	T>C	155410650	snv
4	DCHS2	G>A	155410663	snv
4	DCHS2	G>A	155410667	snv
4	DCHS2	G>A	155410672	snv
4	DCHS2	G>T	155410678	snv
4	DCHS2	G>A	155410727	snv
4	DCHS2	G>C	155410731	snv
4	DCHS2	T>G	155410790	snv
4	DCHS2	G>A	155410822	snv
4	DCHS2	A>G	155410909	snv
4	DCHS2	G>A	155410926	snv
4	DCHS2	G>A	155410986	snv
4	DCHS2	T>C	155411065	snv
4	DCHS2	C>T	155411073	snv
4	DCHS2	G>A	155411089	snv
4	DCHS2	A>C	155411104	snv
4	DCHS2	C>T	155411320	snv
4	DCHS2	G>A	155411341	snv
4	DCHS2	G>T	155411450	snv

4	DCHS2	C>A	155411482	snv
4	DCHS2	C>T	155411517	snv
4	DCHS2	C>T	155411614	snv
4	DCHS2	G>A	155411649	snv
4	DCHS2	G>A	155411772	snv
4	DCHS2	C>T	155411821	snv
4	DCHS2	G>T	155411858	snv
4	DCHS2	A>C	155411939	snv
4	DCHS2	G>A	155412012	snv
4	DCHS2	T>C	155412090	snv
4	DCHS2	G>A	155412430	snv
4	DCHS2	C>G	155412459	snv
4	DCHS2	C>T	155412464	snv
4	NFKB1	T>A	103446697	snv
4	NFKB1	G>A	103488287	snv
4	NFKB1	G>A	103505865	snv
4	NFKB1	G>A	103514641	snv
4	NFKB1	T>C	103514658	snv
4	NFKB1	T>C	103517382	snv
4	NFKB1	G>A	103517393	snv
4	NFKB1	C>T	103517463	snv
4	NFKB1	A>G	103518700	snv
4	NFKB1	A>G	103522068	snv
4	NFKB1	C>T	103522114	snv
4	NFKB1	G>A	103527655	snv
4	NFKB1	T>G	103528817	snv
4	NFKB1	G>C	103533631	snv
4	NFKB1	C>T	103537619	snv
4	DCHS2	A>AGTT	155158259	insertion
4	DCHS2	A>AC	155160424	insertion
5	SLC22A4	G>T	131630497	snv
5	SLC22A4	C>T	131630573	snv
5	SLC22A4	T>C	131663062	snv
5	SLC22A4	C>G	131670546	snv
5	SLC22A4	T>C	131676313	snv
5	SLC22A4	C>T	131676320	snv
5	SLC22A5	T>A	131705723	snv
5	SLC22A5	C>T	131705739	snv
5	SLC22A5	T>C	131705949	snv
5	SLC22A5	C>T	131714106	snv
5	SLC22A5	T>C	131714122	snv
5	SLC22A5	T>C	131714166	snv
5	SLC22A5	A>G	131721174	snv

5	SLC22A5	A>T	131722767	snv
5	SLC22A5	T>C	131726471	snv
5	SLC22A5	A>G	131726578	snv
5	SLC22A5	T>G	131728202	snv
5	SLC22A5	A>G	131728225	snv
5	SLC22A5	C>T	131728291	snv
5	SLC22A5	G>T	131729880	snv
5	SLC22A5	C>T	131729935	snv
6	ARG1	G>A	131894425	snv
6	ARG1	A>G	131894444	snv
6	ARG1	G>C	131900357	snv
6	ARG1	T>C	131903769	snv
6	ARG1	G>A	131903820	snv
6	ARG1	A>C	131904627	snv
6	MAP3K5	ACCGCCG>A	137113183	deletion
6	MAP7	GCTT>G	136681923	deletion
6	HDAC2	T>G	114264531	snv
6	HDAC2	T>A	114267287	snv
6	HDAC2	C>T	114274492	snv
6	HDAC2	T>C	114281189	snv
6	MAP3K5	T>C	136879952	snv
6	MAP3K5	G>A	136904783	snv
6	MAP3K5	G>A	136926387	snv
6	MAP3K5	T>C	136934321	snv
6	MAP3K5	T>C	136944014	snv
6	MAP3K5	A>G	136944063	snv
6	MAP3K5	T>C	136977568	snv
6	MAP3K5	T>C	136980386	snv
6	MAP3K5	G>A	137015311	snv
6	MAP3K5	T>G	137026266	snv
6	MAP3K5	G>A	137113137	snv
6	MAP3K5	G>C	137113248	snv
6	MAP3K5	G>A	137113272	snv
6	MAP7	C>T	136667210	snv
6	MAP7	T>G	136667216	snv
6	MAP7	G>C	136677908	snv
6	MAP7	T>C	136681856	snv
6	MAP7	T>G	136681926	snv
6	MAP7	G>A	136682172	snv
6	MAP7	G>C	136682226	snv
6	MAP7	A>G	136683828	snv
6	MAP7	C>T	136687111	snv
6	MAP7	C>T	136687150	snv

6	MAP7	C>T	136687471	snv
6	MAP7	G>A	136732773	snv
6	SESN1	ACTT>A	109314090	deletion
6	PDE7B	G>A	136429894	snv
6	PDE7B	G>A	136468571	snv
6	PDE7B	G>A	136476782	snv
6	PDE7B	C>T	136476911	snv
6	PDE7B	T>C	136495009	snv
6	PDE7B	A>G	136500215	snv
6	PDE7B	C>G	136512972	snv
6	RSPH3	T>C	159398633	snv
6	RSPH3	C>T	159398700	snv
6	RSPH3	G>A	159398764	snv
6	RSPH3	C>T	159398803	snv
6	RSPH3	T>G	159398835	snv
6	RSPH3	A>G	159399348	snv
6	RSPH3	C>T	159401898	snv
6	RSPH3	G>C	159403651	snv
6	RSPH3	G>C	159407347	snv
6	RSPH3	T>C	159407355	snv
6	RSPH3	C>T	159407415	snv
6	RSPH3	C>T	159407416	snv
6	RSPH3	C>T	159407446	snv
6	RSPH3	G>A	159407447	snv
6	RSPH3	T>C	159414899	snv
6	RSPH3	T>C	159414934	snv
6	RSPH3	G>C	159420545	snv
6	RSPH3	G>A	159420548	snv
6	RSPH3	T>C	159420574	snv
6	RSPH3	C>T	159420772	snv
6	RSPH3	G>T	159420802	snv
6	RSPH3	A>C	159420965	snv
6	SESN1	G>T	109308788	snv
6	SESN1	C>G	109311912	snv
6	SESN1	T>C	109319770	snv
6	SESN1	C>T	109322554	snv
6	SESN1	G>T	109323519	snv
6	SESN1	A>C	109330622	snv
6	SESN1	G>A	109415046	snv
7	CDHR3	TA>T	105635238	deletion
7	CDHR3	A>G	105603809	snv
7	CDHR3	G>A	105615406	snv
7	CDHR3	G>C	105615426	snv

7	CDHR3	T>G	105621500	snv
7	CDHR3	G>T	105621512	snv
7	CDHR3	C>T	105636735	snv
7	CDHR3	C>A	105645063	snv
7	CDHR3	G>T	105645064	snv
7	CDHR3	C>T	105645098	snv
7	CDHR3	G>A	105653450	snv
7	CDHR3	T>G	105653459	snv
7	CDHR3	A>C	105656442	snv
7	CDHR3	G>A	105658451	snv
7	CDHR3	C>G	105658460	snv
7	CDHR3	T>C	105662690	snv
7	CDHR3	C>T	105662809	snv
7	CDHR3	C>A	105662869	snv
7	CDHR3	G>A	105664944	snv
7	CDHR3	T>C	105671267	snv
7	CDHR3	G>A	105673067	snv
7	CDHR3	A>C	105673118	snv
7	FTSJ2	G>A	2275177	snv
7	FTSJ2	G>A	2279284	snv
7	FTSJ2	G>A	2279319	snv
7	FTSJ2	C>A	2281798	snv
7	FTSJ2	A>G	2281803	snv
7	NOM1	AG>A	156743341	deletio n
7	NOM1	AAAG>A	156743028	deletio n
7	MET	G>A	116339209	snv
7	MET	A>T	116339241	snv
7	MET	C>T	116339528	snv
7	MET	C>T	116339672	snv
7	MET	A>G	116340086	snv
7	MET	T>C	116340223	snv
7	MET	C>T	116340251	snv
7	MET	C>T	116340269	snv
7	MET	G>T	116395473	snv
7	MET	C>T	116395478	snv
7	MET	A>G	116397572	snv
7	MET	C>T	116398608	snv
7	MET	C>T	116409777	snv
7	MET	C>T	116409830	snv
7	MET	C>T	116411923	snv
7	MET	T>C	116419011	snv
7	MET	C>T	116422068	snv
7	MET	C>T	116435768	snv

7	MET	G>A	116436022	snv
7	MET	G>A	116436097	snv
7	NOM1	C>A	156742436	snv
7	NOM1	G>T	156742448	snv
7	NOM1	C>T	156742464	snv
7	NOM1	C>G	156742501	snv
7	NOM1	G>A	156742562	snv
7	NOM1	A>G	156742605	snv
7	NOM1	C>T	156742615	snv
7	NOM1	G>A	156742676	snv
7	NOM1	G>A	156742725	snv
7	NOM1	C>T	156742742	snv
7	NOM1	A>C	156742746	snv
7	NOM1	G>A	156742749	snv
7	NOM1	C>A	156742779	snv
7	NOM1	A>C	156742796	snv
7	NOM1	G>C	156742965	snv
7	NOM1	C>A	156743010	snv
7	NOM1	C>T	156743073	snv
7	NOM1	C>G	156743157	snv
7	NOM1	C>A	156743264	snv
7	NOM1	G>A	156745224	snv
7	NOM1	T>C	156746803	snv
7	NOM1	C>T	156746908	snv
7	NOM1	C>G	156746989	snv
7	NOM1	G>A	156752584	snv
7	NOM1	G>A	156752602	snv
7	NOM1	C>T	156752760	snv
7	NOM1	C>T	156754916	snv
7	NOM1	C>G	156755824	snv
7	NOM1	T>C	156755862	snv
7	NOM1	A>T	156756633	snv
7	NOM1	A>G	156756646	snv
7	NOM1	A>G	156759675	snv
7	NOM1	C>T	156761817	snv
7	NOM1	G>A	156761818	snv
7	NOM1	G>A	156761827	snv
7	NOM1	G>C	156762224	snv
7	NOM1	G>A	156762248	snv
7	NOM1	A>G	156762340	snv
7	NOS3	G>C	150692363	snv
7	NOS3	A>G	150693512	snv
7	NOS3	G>A	150693556	snv
7	NOS3	A>T	150693598	snv
7	NOS3	C>T	150693603	snv

7	NOS3	C>G	150693896	snv
7	NOS3	T>C	150695726	snv
7	NOS3	C>T	150696066	snv
7	NOS3	G>A	150696076	snv
7	NOS3	C>T	150696078	snv
7	NOS3	T>G	150696111	snv
7	NOS3	G>A	150698352	snv
7	NOS3	G>A	150700291	snv
7	NOS3	C>T	150700422	snv
7	NOS3	C>G	150700483	snv
7	NOS3	G>A	150704246	snv
7	NOS3	C>G	150704250	snv
7	NOS3	C>T	150704348	snv
7	NOS3	G>A	150707312	snv
7	NOS3	G>T	150707344	snv
7	NOS3	C>T	150707718	snv
7	NOS3	G>A	150707795	snv
7	NOS3	G>A	150707894	snv
7	NOS3	C>T	150708060	snv
7	NOS3	C>T	150709472	snv
7	NOS3	G>A	150710392	snv
7	NOS3	G>A	150710818	snv
7	NOS3	T>C	150710907	snv
7	NOS3	C>A	150711143	snv
7	PKD1L1	T>C	47835733	snv
7	PKD1L1	A>G	47840302	snv
7	PKD1L1	C>G	47840310	snv
7	PKD1L1	G>A	47840336	snv
7	PKD1L1	C>T	47840387	snv
7	PKD1L1	G>A	47840435	snv
7	PKD1L1	A>G	47842894	snv
7	PKD1L1	G>A	47847865	snv
7	PKD1L1	G>A	47849167	snv
7	PKD1L1	C>T	47851623	snv
7	PKD1L1	C>T	47852786	snv
7	PKD1L1	G>C	47852814	snv
7	PKD1L1	C>T	47852837	snv
7	PKD1L1	C>T	47854941	snv
7	PKD1L1	G>A	47854949	snv
7	PKD1L1	C>T	47854956	snv
7	PKD1L1	G>A	47854959	snv
7	PKD1L1	G>A	47854977	snv
7	PKD1L1	C>T	47866960	snv
7	PKD1L1	T>C	47869038	snv
7	PKD1L1	C>A	47872781	snv

7	PKD1L1	A>G	47872845	snv
7	PKD1L1	C>T	47873941	snv
7	PKD1L1	C>T	47873954	snv
7	PKD1L1	G>C	47874579	snv
7	PKD1L1	G>A	47874630	snv
7	PKD1L1	C>T	47874634	snv
7	PKD1L1	G>A	47874839	snv
7	PKD1L1	C>T	47876600	snv
7	PKD1L1	G>A	47879049	snv
7	PKD1L1	C>T	47879080	snv
7	PKD1L1	A>G	47879168	snv
7	PKD1L1	C>T	47884663	snv
7	PKD1L1	T>C	47886583	snv
7	PKD1L1	G>A	47892736	snv
7	PKD1L1	C>T	47898311	snv
7	PKD1L1	A>C	47898389	snv
7	PKD1L1	C>T	47898480	snv
7	PKD1L1	C>T	47913504	snv
7	PKD1L1	G>T	47913560	snv
7	PKD1L1	T>C	47913579	snv
7	PKD1L1	G>A	47913580	snv
7	PKD1L1	C>T	47917087	snv
7	PKD1L1	T>C	47917126	snv
7	PKD1L1	G>A	47920345	snv
7	PKD1L1	A>G	47920371	snv
7	PKD1L1	C>T	47921536	snv
7	PKD1L1	A>G	47921563	snv
7	PKD1L1	G>A	47921640	snv
7	PKD1L1	G>A	47921653	snv
7	PKD1L1	A>T	47921682	snv
7	PKD1L1	G>A	47924233	snv
7	PKD1L1	C>G	47925331	snv
7	PKD1L1	A>G	47925384	snv
7	PKD1L1	G>A	47925424	snv
7	PKD1L1	T>G	47925485	snv
7	PKD1L1	G>A	47925535	snv
7	PKD1L1	G>C	47925562	snv
7	PKD1L1	T>G	47925581	snv
7	PKD1L1	G>A	47925609	snv
7	PKD1L1	C>T	47927649	snv
7	PKD1L1	G>A	47927650	snv
7	PKD1L1	C>T	47927744	snv
7	PKD1L1	C>T	47930148	snv
7	PKD1L1	T>C	47930180	snv
7	PKD1L1	C>T	47930194	snv

7	PKD1L1	A>T	47930277	snv
7	PKD1L1	A>C	47930301	snv
7	PKD1L1	C>T	47933494	snv
7	PKD1L1	T>A	47933652	snv
7	PKD1L1	C>T	47942032	snv
7	PKD1L1	T>C	47942035	snv
7	PKD1L1	T>C	47944756	snv
7	PKD1L1	G>A	47945465	snv
7	PKD1L1	G>A	47947697	snv
7	PKD1L1	C>T	47947752	snv
7	PKD1L1	G>A	47947798	snv
7	PKD1L1	T>C	47947836	snv
7	PKD1L1	A>G	47955049	snv
7	PKD1L1	T>G	47955065	snv
7	PKD1L1	A>G	47955167	snv
7	PKD1L1	G>A	47968830	snv
7	PKD1L1	G>C	47968850	snv
7	PKD1L1	C>A	47968927	snv
7	PKD1L1	A>G	47968928	snv
7	PKD1L1	G>A	47968948	snv
7	PKD1L1	C>G	47969077	snv
7	PKD1L1	C>T	47970746	snv
7	PKD1L1	C>T	47970807	snv
7	PKD1L1	G>A	47970910	snv
7	PKD1L1	C>T	47971562	snv
7	PKD1L1	G>C	47971563	snv
7	PKD1L1	A>G	47971575	snv
7	PKD1L1	G>A	47971626	snv
7	PKD1L1	C>T	47979866	snv
7	PKD1L1	C>T	47983053	snv
8	KLF10	T>G	103663912	snv
8	KLF10	T>C	103663957	snv
8	KLF10	G>C	103663994	snv
8	KLF10	C>A	103664003	snv
8	KLF10	C>T	103664622	snv
8	TOX	C>T	59727990	snv
8	TOX	G>A	59728090	snv
8	TOX	G>A	59750664	snv
8	TOX	C>T	59851984	snv
9	ASS1	C>T	133333853	snv
9	ASS1	G>T	133333936	snv
9	ASS1	C>T	133346226	snv
9	ASS1	A>G	133352282	snv
9	ASS1	C>T	133352335	snv
9	ASS1	G>A	133355180	snv

9	ASS1	C>T	133355802	snv
9	ASS1	G>A	133355834	snv
9	ASS1	T>C	133364757	snv
9	ASS1	C>T	133364791	snv
9	ASS1	C>T	133374898	snv
9	KLF4	C>T	110248068	snv
9	KLF4	G>A	110249316	snv
9	KLF4	T>C	110249415	snv
9	KLF4	G>C	110249590	snv
9	KLF4	C>G	110249640	snv
9	KLF4	G>T	110249658	snv
9	KLF4	A>G	110249668	snv
9	KLF4	G>A	110249702	snv
9	KLF4	C>T	110249891	snv
9	KLF4	G>C	110249896	snv
9	KLF4	G>A	110250058	snv
9	KLF4	G>A	110250171	snv
9	KLF4	T>C	110251215	snv
10	CYP2C9	A>T	96701758	snv
10	CYP2C9	G>T	96702007	snv
10	CYP2C9	G>A	96702066	snv
10	CYP2C9	A>G	96708974	snv
10	CYP2C9	T>C	96740958	snv
10	CYP2C9	C>G	96741058	snv
10	CYP2C9	C>T	96748635	snv
10	CYP2C9	C>T	96748701	snv
10	CYP2C9	A>G	96748733	snv
10	CYP2C9	GA>G	96709038	deletion
10	CYP2E1	G>A	135341013	snv
10	CYP2E1	G>A	135342101	snv
10	CYP2E1	G>A	135345129	snv
10	CYP2E1	C>A	135345234	snv
10	CYP2E1	G>A	135345675	snv
10	CYP2E1	T>C	135345752	snv
10	CYP2E1	G>A	135346271	snv
10	CYP2E1	T>C	135347397	snv
10	CYP2E1	G>A	135350592	snv
10	CYP2E1	C>G	135350643	snv
10	CYP2E1	G>C	135351285	snv
10	CYP2E1	A>G	135351352	snv
10	CYP2E1	T>C	135351362	snv
10	CYP2E1	A>T	135352356	snv
10	NFKB2	ACGGGTATGGGTGCAGGGGGTGGGT>A	104157162	deletion

10	DOCK1	T>C	128776246	snv
10	DOCK1	G>A	128795081	snv
10	DOCK1	A>C	128798524	snv
10	DOCK1	A>C	128807018	snv
10	DOCK1	C>G	128810554	snv
10	DOCK1	T>C	128821486	snv
10	DOCK1	A>G	128830409	snv
10	DOCK1	C>T	128830453	snv
10	DOCK1	G>A	128830454	snv
10	DOCK1	A>G	128904519	snv
10	DOCK1	A>G	128908616	snv
10	DOCK1	C>G	128923768	snv
10	DOCK1	A>G	128926013	snv
10	DOCK1	G>A	129160362	snv
10	DOCK1	G>A	129160458	snv
10	DOCK1	G>A	129172328	snv
10	DOCK1	C>T	129172355	snv
10	DOCK1	T>C	129178348	snv
10	DOCK1	C>T	129202636	snv
10	DOCK1	C>T	129207417	snv
10	DOCK1	C>T	129207429	snv
10	DOCK1	T>C	129213442	snv
10	DOCK1	A>C	129213451	snv
10	DOCK1	T>C	129216658	snv
10	DOCK1	C>T	129216718	snv
10	DOCK1	C>T	129216763	snv
10	DOCK1	A>G	129216772	snv
10	DOCK1	C>T	129224206	snv
10	DOCK1	G>A	129224207	snv
10	DOCK1	C>A	129231579	snv
10	DOCK1	C>T	129237488	snv
10	DOCK1	G>A	129242435	snv
10	DOCK1	C>T	129242446	snv
10	DOCK1	G>A	129242461	snv
10	DOCK1	G>A	129242473	snv
10	DOCK1	G>A	129242515	snv
10	DOCK1	G>A	129245684	snv
10	DOCK1	G>A	129249662	snv
10	DOCK1	C>T	129249676	snv
10	LDB1	G>C	103868817	snv
10	LDB1	C>T	103870346	snv
10	NFKB2	G>A	104156028	snv
10	NFKB2	G>A	104157812	snv
10	NFKB2	A>G	104159196	snv
10	NFKB2	C>T	104159245	snv

10	NFKB2	C>A	104160058	snv
10	NFKB2	A>G	104160434	snv
10	NFKB2	G>A	104160482	snv
10	NFKB2	C>G	104160566	snv
10	NFKB2	A>T	104160739	snv
10	SEC31B	C>A	102247408	snv
10	SEC31B	T>C	102247524	snv
10	SEC31B	G>C	102247526	snv
10	SEC31B	C>T	102247821	snv
10	SEC31B	C>A	102247837	snv
10	SEC31B	G>A	102249889	snv
10	SEC31B	G>T	102249946	snv
10	SEC31B	C>T	102256058	snv
10	SEC31B	G>A	102256120	snv
10	SEC31B	A>G	102256169	snv
10	SEC31B	C>T	102256188	snv
10	SEC31B	C>T	102257820	snv
10	SEC31B	G>A	102257821	snv
10	SEC31B	C>T	102258478	snv
10	SEC31B	T>G	102258922	snv
10	SEC31B	C>T	102258991	snv
10	SEC31B	C>G	102259333	snv
10	SEC31B	G>A	102262181	snv
10	SEC31B	C>G	102262227	snv
10	SEC31B	G>T	102265155	snv
10	SEC31B	G>A	102265183	snv
10	SEC31B	A>G	102265815	snv
10	SEC31B	C>A	102265826	snv
10	SEC31B	A>C	102265847	snv
10	SEC31B	C>T	102267211	snv
10	SEC31B	G>C	102267763	snv
10	SEC31B	C>T	102267771	snv
10	SEC31B	C>A	102269085	snv
10	SEC31B	T>C	102269173	snv
10	SEC31B	A>C	102269182	snv
10	SEC31B	A>G	102269206	snv
10	SEC31B	C>A	102275968	snv
11	ARHGAP1	G>A	46700663	snv
11	ARHGAP1	C>T	46701765	snv
11	ARHGAP1	A>G	46702059	snv
11	ARHGAP1	G>T	46702225	snv
11	ARHGAP1	G>A	46702608	snv
11	ARHGAP1	T>C	46702920	snv
11	CAT	G>A	34477567	snv
11	CAT	C>T	34482908	snv

11	CAT	C>T	34485711	snv
11	CAT	C>T	34492546	snv
11	CAT	T>C	34492548	snv
11	OR51B5	CCAGCCCCAGGTCTGTGG>C	5364541	deletion
11	OR51B5	G>A	5364097	snv
11	OR51B5	C>T	5364174	snv
11	OR51B5	G>A	5364220	snv
11	OR51B5	G>A	5364276	snv
11	OR51B5	C>T	5364295	snv
11	OR51B5	G>C	5364296	snv
11	OR51B5	G>A	5364431	snv
11	OR51B5	A>G	5364450	snv
11	OR51B5	C>T	5364471	snv
11	OR51B5	C>T	5364476	snv
11	OR51B5	T>C	5364493	snv
11	OR51B5	G>T	5364522	snv
11	OR51B5	C>T	5364742	snv
11	OR51B6	A>C	5372751	snv
11	OR51B6	A>G	5372856	snv
11	OR51B6	T>A	5372863	snv
11	OR51B6	G>A	5372881	snv
11	OR51B6	T>C	5373006	snv
11	OR51B6	T>C	5373013	snv
11	OR51B6	T>C	5373095	snv
11	OR51B6	A>G	5373104	snv
11	OR51B6	G>A	5373111	snv
11	OR51B6	G>A	5373114	snv
11	OR51B6	C>T	5373129	snv
11	OR51B6	C>G	5373170	snv
11	OR51B6	T>G	5373242	snv
11	OR51B6	C>T	5373251	snv
11	OR51B6	C>A	5373309	snv
11	OR51B6	T>C	5373311	snv
11	OR51B6	A>G	5373334	snv
11	OR51B6	G>C	5373497	snv
11	OR51B6	C>A	5373562	snv
11	OR51B6	C>T	5373575	snv
11	OR51B6	C>T	5373644	snv
11	OR51B6	T>C	5373646	snv
11	SOX6	C>T	16007938	snv
11	SOX6	G>A	16010547	snv
11	SOX6	C>T	16071294	snv
11	SOX6	A>G	16133413	snv
11	SOX6	C>T	16208386	snv

11	SOX6	C>T	16362581	snv
12	KRT80	G>A	52565198	snv
12	KRT80	G>A	52565207	snv
12	KRT80	G>T	52565322	snv
12	KRT80	G>A	52566047	snv
12	KRT80	G>T	52566070	snv
12	KRT80	G>C	52566882	snv
12	KRT80	C>T	52567434	snv
12	KRT80	G>A	52567488	snv
12	KRT80	C>T	52567503	snv
12	KRT80	G>C	52567527	snv
12	KRT80	G>A	52579200	snv
12	KRT80	G>A	52579242	snv
12	KRT80	C>T	52579293	snv
12	KRT80	G>A	52579294	snv
12	KRT80	G>T	52585488	snv
12	KRT80	G>A	52585628	snv
12	NOS1	C>G	117655876	snv
12	NOS1	G>T	117657991	snv
12	NOS1	A>G	117665264	snv
12	NOS1	G>A	117665343	snv
12	NOS1	G>T	117665377	snv
12	NOS1	G>A	117665399	snv
12	NOS1	G>A	117669866	snv
12	NOS1	G>A	117669914	snv
12	NOS1	T>C	117672412	snv
12	NOS1	G>A	117672446	snv
12	NOS1	G>C	117672539	snv
12	NOS1	G>A	117672548	snv
12	NOS1	G>A	117685255	snv
12	NOS1	G>A	117685270	snv
12	NOS1	G>A	117691490	snv
12	NOS1	T>C	117693792	snv
12	NOS1	G>A	117693817	snv
12	NOS1	A>G	117701714	snv
12	NOS1	T>C	117701743	snv
12	NOS1	G>A	117718593	snv
12	NOS1	G>A	117723117	snv
12	NOS1	G>A	117749361	snv
12	NOS1	G>A	117768337	snv
12	NOS1	G>A	117768411	snv
12	NOS1	G>A	117768417	snv
12	NOS1	C>T	117768455	snv
12	NOS1	C>T	117768574	snv
12	NOS1	G>A	117768602	snv

12	SLCO1A2	G>C	21422492	snv
12	SLCO1A2	G>A	21428307	snv
12	SLCO1A2	G>C	21445227	snv
12	SLCO1A2	T>C	21446936	snv
12	SLCO1A2	G>A	21448588	snv
12	SLCO1A2	G>T	21448604	snv
12	SLCO1A2	G>A	21453424	snv
12	SLCO1A2	G>A	21453466	snv
12	SLCO1A2	T>G	21457434	snv
12	SLCO1A2	T>A	21459876	snv
12	SLCO1A2	A>G	21467546	snv
12	SLCO1A2	A>G	21471732	snv
12	SLCO1A2	A>G	21487544	snv
12	SLCO1B1	A>G	21329738	snv
12	SLCO1B1	G>A	21329761	snv
12	SLCO1B1	C>A	21329813	snv
12	SLCO1B1	T>C	21331549	snv
12	SLCO1B1	T>C	21331599	snv
12	SLCO1B1	C>T	21331625	snv
12	SLCO1B1	A>G	21331891	snv
12	SLCO1B1	A>G	21349885	snv
12	SLCO1B1	G>A	21350034	snv
12	SLCO1B1	C>T	21353557	snv
12	SLCO1B1	C>G	21355489	snv
12	SLCO1B1	G>A	21355537	snv
12	SLCO1B1	C>T	21355597	snv
12	SLCO1B1	C>T	21358922	snv
12	SLCO1B1	G>C	21358933	snv
12	SLCO1B1	A>G	21358965	snv
12	SLCO1B1	A>C	21391976	snv
12	SLCO1B3	A>G	20968683	snv
12	SLCO1B3	C>T	20968741	snv
12	SLCO1B3	C>G	21007985	snv
12	SLCO1B3	A>G	21008031	snv
12	SLCO1B3	A>G	21011421	snv
12	SLCO1B3	C>A	21011463	snv
12	SLCO1B3	T>G	21011480	snv
12	SLCO1B3	A>G	21014030	snv
12	SLCO1B3	G>A	21014063	snv
12	SLCO1B3	A>G	21015706	snv
12	SLCO1B3	G>A	21015707	snv
12	SLCO1B3	C>G	21015737	snv
12	SLCO1B3	G>A	21015760	snv
12	SLCO1B3	C>G	21028186	snv
12	SLCO1B3	T>A	21028200	snv

12	SLCO1B3	A>G	21036411	snv
12	SLCO1B3	T>C	21036468	snv
12	SLCO1B3	T>C	21036533	snv
12	SLCO1B3	G>A	21054369	snv
12	SLCO1B3	T>C	21054373	snv
12	SLCO1B3	C>T	21069048	snv
12	SLCO1B3	G>A	21069049	snv
12	SUDS3	T>C	118818019	snv
12	SLCO1B3	A>AT	21028266	insertion
13	FLT1	G>A	28883002	snv
13	FLT1	G>A	28883061	snv
13	FLT1	A>G	28893642	snv
13	FLT1	C>T	28908193	snv
13	FLT1	G>T	28908265	snv
13	FLT1	G>A	28919682	snv
13	FLT1	G>A	28959077	snv
13	FLT1	C>T	28963765	snv
13	FLT1	G>A	28979994	snv
13	FLT1	C>T	28980020	snv
13	FLT1	G>A	29001374	snv
13	FLT1	G>A	29001430	snv
13	FLT1	C>T	29004277	snv
13	FLT1	G>A	29012466	snv
13	FLT1	A>G	29041155	snv
13	FLT1	C>T	29068965	snv
13	FLT1	G>A	29068969	snv
13	N4BP2L2	G>T	33016603	snv
13	N4BP2L2	C>T	33016660	snv
13	N4BP2L2	T>C	33016819	snv
13	N4BP2L2	T>G	33016930	snv
13	N4BP2L2	T>C	33017043	snv
13	N4BP2L2	G>T	33017158	snv
13	N4BP2L2	C>A	33017909	snv
13	N4BP2L2	T>A	33017937	snv
13	N4BP2L2	T>G	33017943	snv
13	N4BP2L2	C>A	33018239	snv
13	N4BP2L2	C>T	33101574	snv
13	N4BP2L2	A>G	33110351	snv
13	N4BP2L2	C>G	33110361	snv
13	N4BP2L2	C>A	33110539	snv
13	N4BP2L2	C>T	33110739	snv
13	N4BP2L2	T>C	33110815	snv
13	N4BP2L2	C>T	33110831	snv
13	N4BP2L2	A>G	33110898	snv

13	N4BP2L2	C>G	33111069	snv
13	RNF113B	C>T	98828816	snv
13	RNF113B	T>C	98828892	snv
13	RNF113B	C>T	98828928	snv
13	RNF113B	G>T	98828990	snv
13	RNF113B	T>C	98829176	snv
13	RNF113B	C>T	98829217	snv
13	RNF113B	C>G	98829266	snv
14	ARG2	G>A	68086802	snv
14	ARG2	G>C	68113738	snv
14	ARG2	G>A	68117496	snv
14	ARG2	G>A	68117524	snv
14	ARG2	C>T	68117623	snv
14	EML1	C>T	100204181	snv
14	EML1	C>T	100317272	snv
14	EML1	T>C	100331876	snv
14	EML1	G>A	100331926	snv
14	EML1	C>T	100331945	snv
14	EML1	C>G	100361072	snv
14	EML1	C>T	100374013	snv
14	EML1	T>C	100374019	snv
14	EML1	C>T	100375707	snv
14	EML1	C>T	100376626	snv
14	EML1	T>C	100380948	snv
14	EML1	C>T	100380995	snv
14	EML1	G>A	100384160	snv
14	EML1	G>A	100402445	snv
14	EML1	C>G	100405562	snv
14	EML1	C>T	100405654	snv
14	PAPLN	A>G	73711394	snv
14	PAPLN	C>T	73712808	snv
14	PAPLN	G>A	73716774	snv
14	PAPLN	C>A	73717669	snv
14	PAPLN	G>A	73717720	snv
14	PAPLN	C>T	73717721	snv
14	PAPLN	G>T	73718474	snv
14	PAPLN	G>A	73718513	snv
14	PAPLN	G>A	73718514	snv
14	PAPLN	C>T	73718525	snv
14	PAPLN	A>C	73719455	snv
14	PAPLN	G>A	73721245	snv
14	PAPLN	C>T	73721300	snv
14	PAPLN	C>T	73721635	snv
14	PAPLN	C>A	73721705	snv
14	PAPLN	C>T	73726042	snv

14	PAPLN	C>T	73726132	snv
14	PAPLN	A>G	73726151	snv
14	PAPLN	C>T	73726194	snv
14	PAPLN	G>A	73727448	snv
14	PAPLN	G>A	73727483	snv
14	PAPLN	T>G	73727509	snv
14	PAPLN	C>T	73729246	snv
14	PAPLN	C>T	73729347	snv
14	PAPLN	G>A	73729498	snv
14	PAPLN	C>T	73730439	snv
14	PAPLN	G>A	73730935	snv
14	PAPLN	C>T	73731012	snv
14	PAPLN	G>A	73731310	snv
14	PAPLN	C>T	73731313	snv
14	PAPLN	C>T	73732103	snv
14	PAPLN	G>A	73732154	snv
14	PAPLN	C>G	73732212	snv
14	PAPLN	G>A	73733285	snv
14	PAPLN	G>T	73733432	snv
14	PAPLN	C>T	73735366	snv
14	PAPLN	C>T	73739217	snv
14	PAPLN	G>A	73739369	snv
14	SALL2	C>G	21990252	snv
14	SALL2	G>T	21990982	snv
14	SALL2	C>G	21991144	snv
14	SALL2	G>C	21991343	snv
14	SALL2	T>C	21991411	snv
14	SALL2	G>A	21991589	snv
14	SALL2	C>G	21991626	snv
14	SALL2	C>T	21991630	snv
14	SALL2	G>A	21991691	snv
14	SALL2	C>A	21991697	snv
14	SALL2	A>G	21991804	snv
14	SALL2	G>A	21991990	snv
14	SALL2	T>C	21992057	snv
14	SALL2	C>T	21992272	snv
14	SALL2	T>C	21992397	snv
14	SALL2	G>A	21992410	snv
14	SALL2	G>A	21992629	snv
14	SALL2	C>T	21992841	snv
14	SALL2	G>C	21993200	snv
14	SALL2	G>A	21993498	snv
14	SALL2	G>C	21993564	snv
14	SALL2	G>C	21993638	snv
14	SALL2	C>T	21994198	snv

14	SALL2	C>T	21994239	snv
15	AQP9	C>T	58465385	snv
15	AQP9	C>T	58471368	snv
15	AQP9	G>C	58471530	snv
15	AQP9	T>C	58476238	snv
15	AQP9	A>G	58476281	snv
15	SIN3A	G>A	75664404	snv
15	SIN3A	T>A	75668131	snv
15	SIN3A	G>A	75684695	snv
15	SIN3A	A>G	75688670	snv
15	SIN3A	A>G	75694291	snv
15	SIN3A	T>C	75702496	snv
15	SIN3A	G>A	75703899	snv
15	SIN3A	T>C	75703979	snv
16	MAPK3	C>T	30128167	snv
16	MAPK3	G>A	30129103	snv
17	MARCH10	G>C	60778992	snv
17	MARCH10	A>G	60779025	snv
17	MARCH10	A>G	60782904	snv
17	MARCH10	G>C	60782924	snv
17	MARCH10	C>T	60802418	snv
17	MARCH10	C>G	60813382	snv
17	MARCH10	C>T	60813470	snv
17	MARCH10	G>A	60813627	snv
17	MARCH10	G>A	60813751	snv
17	MARCH10	G>A	60813765	snv
17	MARCH10	G>A	60813944	snv
17	MARCH10	A>G	60813954	snv
17	MARCH10	T>C	60813982	snv
17	MARCH10	A>G	60814273	snv
17	MARCH10	G>A	60814313	snv
17	MARCH10	T>C	60814428	snv
17	MARCH10	T>G	60814504	snv
17	MARCH10	C>T	60814560	snv
17	MARCH10	G>A	60814612	snv
17	MARCH10	G>T	60814659	snv
17	MARCH10	G>A	60821773	snv
17	MARCH10	C>T	60837208	snv
17	MARCH10	T>C	60837269	snv
17	MARCH10	G>A	60837337	snv
17	MPO	G>A	56348068	snv
17	MPO	T>C	56348106	snv
17	MPO	G>A	56348154	snv
17	MPO	C>G	56348208	snv
17	MPO	G>A	56349029	snv

17	MPO	G>A	56349102	snv
17	MPO	A>G	56349207	snv
17	MPO	G>A	56350829	snv
17	MPO	C>T	56350986	snv
17	MPO	G>A	56351019	snv
17	MPO	G>A	56356457	snv
17	MPO	T>G	56356532	snv
17	MPO	C>T	56357741	snv
17	MPO	C>A	56357818	snv
17	MYBBP1A	G>A	4442770	snv
17	MYBBP1A	G>A	4442798	snv
17	MYBBP1A	T>C	4442801	snv
17	MYBBP1A	G>A	4442818	snv
17	MYBBP1A	G>A	4442931	snv
17	MYBBP1A	C>T	4443021	snv
17	MYBBP1A	G>A	4443205	snv
17	MYBBP1A	C>T	4443680	snv
17	MYBBP1A	G>A	4443681	snv
17	MYBBP1A	C>G	4443710	snv
17	MYBBP1A	C>G	4443750	snv
17	MYBBP1A	C>T	4444850	snv
17	MYBBP1A	G>A	4445082	snv
17	MYBBP1A	A>T	4445939	snv
17	MYBBP1A	T>G	4446227	snv
17	MYBBP1A	C>T	4446362	snv
17	MYBBP1A	G>A	4446457	snv
17	MYBBP1A	G>A	4447794	snv
17	MYBBP1A	C>T	4447795	snv
17	MYBBP1A	G>T	4447895	snv
17	MYBBP1A	C>T	4447909	snv
17	MYBBP1A	C>G	4447941	snv
17	MYBBP1A	G>T	4448049	snv
17	MYBBP1A	G>A	4448148	snv
17	MYBBP1A	C>T	4448324	snv
17	MYBBP1A	C>T	4448380	snv
17	MYBBP1A	C>T	4448590	snv
17	MYBBP1A	C>T	4448929	snv
17	MYBBP1A	G>A	4448940	snv
17	MYBBP1A	T>C	4449150	snv
17	MYBBP1A	C>T	4449161	snv
17	MYBBP1A	G>A	4451316	snv
17	MYBBP1A	G>A	4451462	snv
17	MYBBP1A	G>A	4451518	snv
17	MYBBP1A	T>C	4451576	snv
17	MYBBP1A	A>G	4451825	snv

17	MYBBP1A	A>C	4451826	snv
17	MYBBP1A	T>C	4451882	snv
17	MYBBP1A	G>A	4452694	snv
17	MYBBP1A	G>A	4453409	snv
17	MYBBP1A	G>C	4453437	snv
17	MYBBP1A	C>T	4453439	snv
17	MYBBP1A	C>T	4453508	snv
17	MYBBP1A	G>A	4455214	snv
17	MYBBP1A	T>C	4455500	snv
17	MYBBP1A	C>T	4455577	snv
17	MYBBP1A	G>A	4457111	snv
17	MYBBP1A	T>C	4457116	snv
17	MYBBP1A	G>C	4457134	snv
17	MYBBP1A	C>T	4457571	snv
17	MYBBP1A	G>T	4458515	snv
17	MYBBP1A	T>C	4458536	snv
17	MYBBP1A	G>T	4458560	snv
17	MYBBP1A	G>A	4458594	snv
17	MYBBP1A	G>C	4458598	snv
17	NOS2	G>A	26085931	snv
17	NOS2	G>C	26086063	snv
17	NOS2	C>T	26086079	snv
17	NOS2	G>T	26087065	snv
17	NOS2	G>C	26087767	snv
17	NOS2	T>C	26089867	snv
17	NOS2	G>T	26089996	snv
17	NOS2	T>G	26091048	snv
17	NOS2	A>G	26092631	snv
17	NOS2	G>A	26092644	snv
17	NOS2	C>T	26092740	snv
17	NOS2	T>C	26094796	snv
17	NOS2	G>A	26096597	snv
17	NOS2	C>T	26097972	snv
17	NOS2	G>A	26099383	snv
17	NOS2	A>G	26105725	snv
17	NOS2	G>A	26105932	snv
17	NOS2	G>A	26107840	snv
17	NOS2	C>T	26107895	snv
17	NOS2	C>T	26108076	snv
17	NOS2	G>A	26109102	snv
17	NOS2	T>C	26109117	snv
17	NOS2	G>A	26110055	snv
17	NOS2	T>C	26115949	snv
18	SLC14A1	C>T	43307246	snv
18	SLC14A1	C>A	43307338	snv

18	SLC14A1	C>G	43307359	snv
18	SLC14A1	G>A	43310313	snv
18	SLC14A1	G>A	43310415	snv
18	SLC14A1	G>A	43311054	snv
18	SLC14A1	G>A	43311131	snv
18	SLC14A1	T>C	43314299	snv
18	SLC14A1	T>C	43316461	snv
18	SLC14A1	A>G	43316538	snv
18	SLC14A1	G>A	43319274	snv
18	SLC14A1	G>A	43319519	snv
18	SLC14A1	A>T	43329864	snv
18	SLC14A1	A>G	43332170	snv
18	SLC14A2	C>T	43204692	snv
18	SLC14A2	G>A	43204734	snv
18	SLC14A2	C>T	43205793	snv
18	SLC14A2	A>G	43206985	snv
18	SLC14A2	C>T	43212329	snv
18	SLC14A2	C>T	43219773	snv
18	SLC14A2	G>A	43224101	snv
18	SLC14A2	G>A	43224125	snv
18	SLC14A2	A>G	43246126	snv
18	SLC14A2	G>A	43246156	snv
18	SLC14A2	C>G	43247001	snv
18	SLC14A2	G>A	43247029	snv
18	SLC14A2	C>A	43249385	snv
18	SLC14A2	G>A	43252883	snv
18	SLC14A2	G>A	43262359	snv
19	COPE	T>C	19011244	snv
19	COPE	C>T	19014109	snv
19	COPE	G>A	19014110	snv
19	COPE	T>C	19016701	snv
19	COPE	G>A	19016722	snv
19	COPE	G>A	19017814	snv
19	COPE	G>T	19017828	snv
19	COPE	A>G	19017833	snv
19	COPE	G>A	19017862	snv
19	COPE	G>A	19017906	snv
19	COPE	C>T	19021816	snv
19	COPE	G>A	19021842	snv
19	COPE	T>C	19023853	snv
19	COPE	G>A	19030083	snv
19	COPE	G>C	19030107	snv
19	COPE	G>C	19030120	snv
19	RHPN2	T>C	33486941	snv
19	RHPN2	C>T	33486950	snv

19	RHPN2	G>A	33486990	snv
19	RHPN2	A>C	33486992	snv
19	RHPN2	G>A	33487052	snv
19	RHPN2	G>C	33487071	snv
19	RHPN2	T>C	33490566	snv
19	RHPN2	G>A	33490585	snv
19	RHPN2	C>G	33493234	snv
19	RHPN2	C>T	33493262	snv
19	RHPN2	A>C	33493744	snv
19	RHPN2	G>A	33493778	snv
19	RHPN2	C>T	33493835	snv
19	RHPN2	G>A	33498967	snv
19	RHPN2	A>G	33498978	snv
19	RHPN2	G>A	33499071	snv
19	RHPN2	A>G	33503562	snv
19	RHPN2	G>A	33512501	snv
19	RHPN2	G>A	33512508	snv
19	RHPN2	C>T	33517515	snv
19	RHPN2	G>A	33535247	snv
19	RHPN2	A>G	33555757	snv
20	OPRL1	C>T	62724091	snv
20	OPRL1	C>T	62724247	snv
20	OPRL1	C>T	62729247	snv
20	OPRL1	G>A	62729272	snv
20	OPRL1	G>A	62729284	snv
20	OPRL1	C>T	62729431	snv
20	OPRL1	C>T	62729464	snv
20	OPRL1	G>A	62729727	snv
20	OPRL1	A>G	62729843	snv
20	OPRL1	C>T	62730003	snv
20	OPRL1	C>T	62730065	snv
20	OPRL1	C>T	62730139	snv
22	CYP2D6	A>G	42523943	snv
22	CYP2D6	G>A	42525756	snv
22	CYP2D6	G>A	42525772	snv
22	CYP2D6	G>A	42526694	snv
22	CYP2D6	A>G	42524323	snv
22	CYP2D6	T>G	42523858	snv
22	CYP2D6	C>A	42524191	snv
22	CYP2D6	G>T	42524219	snv
22	CYP2D6	C>T	42526776	snv
22	CYP2D6	G>GCA	42523532	insertion
22	CYP2D6	G>A	42526644	snv
22	CYP2D6	C>T	42523805	snv

22	CYP2D6	G>A	42523592	snv
22	CYP2D6	A>G	42522600	snv
22	CYP2D6	G>T	42525122	snv
22	CYP2D6	C>A	42525185	snv
22	CYP2D6	C>A	42524202	insertion
22	CYP2D6	G>A	42525036	snv
22	CYP2D6	G>C	42522613	snv
22	CYP2D6	C>T	42523505	snv
22	CYP2D6	C>T	42523514	snv
22	CYP2D6	A>G	42524795	snv
22	CYP2D6	G>C	42525132	snv
22	CYP2D6	A>G	42523539	snv
22	CYP2D6	G>T	42524218	snv
22	CYP2D6	C>T	42525077	snv
22	CYP2D6	C>T	42523610	snv
22	CYP2D6	C>T	42525134	snv
22	CYP2D6	C>T	42526717	snv
22	CYP2D6	C>T	42522916	snv
22	CYP2D6	C>T	42522965	snv
22	CYP2D6	C>T	42522717	snv
22	CYP2D6	C>T	42523528	snv
22	HMOX1	G>C	35777185	snv
22	HMOX1	G>A	35782871	snv
22	HMOX1	C>T	35782911	snv
22	HMOX1	G>A	35782962	snv
22	HMOX1	A>G	35783136	snv
22	HMOX1	C>T	35785887	snv
22	HMOX1	T>C	35789465	snv
22	HMOX1	A>G	35789564	snv
22	MAPK1	G>A	22123601	snv
22	MAPK1	A>T	22127222	snv
22	MAPK1	A>G	22127260	snv
22	MAPK1	T>C	22142580	snv
22	MAPK1	G>A	22160151	snv
22	MAPK1	A>G	22162126	snv
22	MAPK1	C>A	22221665	snv
22	MAPK1	C>T	22221680	snv
22	CYP2D6	G>C	42525798	snv
22	CYP2D6	T>C	42525811	snv
X	EGFL6	G>A	13612963	snv
X	EGFL6	C>T	13612989	snv
X	EGFL6	C>T	13618120	snv
X	EGFL6	A>G	13618137	snv
X	EGFL6	C>T	13618161	snv

X	EGFL6	G>A	13618164	snv
X	EGFL6	T>G	13621476	snv
X	EGFL6	C>T	13621525	snv
X	EGFL6	C>T	13624595	snv
X	EGFL6	C>T	13635937	snv
X	EGFL6	T>C	13645181	snv
X	EGFL6	G>T	13645368	snv
X	EGFL6	G>A	13651155	snv
X	GATA1	G>T	48649571	snv
X	GATA1	G>A	48649717	snv
X	GATA1	A>G	48652271	snv
X	GATA1	G>A	48652502	snv
X	GATA1	G>A	48652572	snv
X	GPM6B	A>G	13794363	snv
X	GPM6B	G>A	13797938	snv
X	GPM6B	C>A	13803848	snv
