# Supplementary material for: Are Genetic Modifiers the Answer to Different Responses to Hydroxyurea Treatment?—A Pharmacogenetic Study in Sickle Cell Anemia Angolan Children
Source: Int J Mol Sci. 2023 May 15;24(10):8792. doi: 10.3390/ijms24108792 (PMC10218819; doi:10.3390/ijms24108792)
Supplement: Supplementary file 1 [file ijms-24-08792-s001.zip › ijms-2316219-supplementary/Supplementary Table S2.pdf]

Table S2. List of genes selected for sequencing (Reference genome h38).

**Gene**	**Chromosome**	**Amplicon Start**	**Amplicon End**
ADAR	chr1	154580467	154580482
ADAR	chr1	154561844	154561938
ADAR	chr1	154558228	154558341
ADAR	chr1	154561026	154561149
ADAR	chr1	154557692	154557820
ADAR	chr1	154560600	154560734
ADAR	chr1	154569598	154569743
ADAR	chr1	154570303	154570452
ADAR	chr1	154562232	154562404
ADAR	chr1	154558656	154558839
ADAR	chr1	154570877	154571061
ADAR	chr1	154569280	154569471
ADAR	chr1	154562659	154562885
ADAR	chr1	154557284	154557519
ADAR	chr1	154573516	154575102
ADCY10	chr1	167871253	167871292
ADCY10	chr1	167844368	167844424
ADCY10	chr1	167806486	167806556
ADCY10	chr1	167849352	167849429
ADCY10	chr1	167829044	167829131
ADCY10	chr1	167863090	167863179
ADCY10	chr1	167793713	167793810
ADCY10	chr1	167865832	167865929
ADCY10	chr1	167873124	167873229
ADCY10	chr1	167803207	167803315
ADCY10	chr1	167792245	167792361
ADCY10	chr1	167791261	167791379
ADCY10	chr1	167849728	167849847
ADCY10	chr1	167817598	167817727
ADCY10	chr1	167823590	167823727
ADCY10	chr1	167839494	167839648
ADCY10	chr1	167798504	167798661
ADCY10	chr1	167778917	167779076
ADCY10	chr1	167870899	167871059
ADCY10	chr1	167802224	167802400
ADCY10	chr1	167847683	167847873
ADCY10	chr1	167852674	167852866
ADCY10	chr1	167787309	167787505
ADCY10	chr1	167779961	167780150
ADCY10	chr1	167830108	167830301
ADCY10	chr1	167793888	167794093
ADCY10	chr1	167868660	167868866

ADCY10	chr1	167874159	167874378
ADCY10	chr1	167805546	167805778
ADCY10	chr1	167825402	167825677
ADCY10	chr1	167815222	167815501
ADCY10	chr1	167814800	167815091
AQP9	chr15	58430764	58430875
AQP9	chr15	58467116	58467235
AQP9	chr15	58458871	58458998
AQP9	chr15	58465266	58465404
AQP9	chr15	58476159	58476331
AQP9	chr15	58471326	58471544
ARG1	chr6	131894422	131894479
ARG1	chr6	131897802	131897875
ARG1	chr6	131903760	131903855
ARG1	chr6	131904199	131904304
ARG1	chr6	131904494	131904631
ARG1	chr6	131902358	131902518
ARG1	chr6	131904881	131905045
ARG1	chr6	131900226	131900425
ARG2	chr14	68087610	68087683
ARG2	chr14	68113360	68113455
ARG2	chr14	68113637	68113742
ARG2	chr14	68086694	68086805
ARG2	chr14	68114763	68114900
ARG2	chr14	68112359	68112519
ARG2	chr14	68108902	68109080
ARG2	chr14	68117431	68117634
ARHGAP1	chr11	46702036	46702113
ARHGAP1	chr11	46701754	46701832
ARHGAP1	chr11	46702842	46702929
ARHGAP1	chr11	46709722	46709810
ARHGAP1	chr11	46717209	46717305
ARHGAP1	chr11	46702560	46702659
ARHGAP1	chr11	46700914	46701018
ARHGAP1	chr11	46702189	46702297
ARHGAP1	chr11	46701197	46701326
ARHGAP1	chr11	46703600	46703732
ARHGAP1	chr11	46717524	46717657
ARHGAP1	chr11	46700589	46700775
ASS1	chr9	133346871	133346902
ASS1	chr9	133376362	133376405
ASS1	chr9	133339497	133339554
ASS1	chr9	133355771	133355836
ASS1	chr9	133374891	133374957
ASS1	chr9	133329691	133329760

ASS1	chr9	133346220	133346291
ASS1	chr9	133342111	133342186
ASS1	chr9	133355102	133355187
ASS1	chr9	133352257	133352348
ASS1	chr9	133327615	133327720
ASS1	chr9	133364719	133364851
ASS1	chr9	133370253	133370410
ASS1	chr9	133333787	133333976
CAT	chr11	34492914	34492977
CAT	chr11	34460560	34460626
CAT	chr11	34492504	34492588
CAT	chr11	34474636	34474741
CAT	chr11	34489834	34489942
CAT	chr11	34472534	34472645
CAT	chr11	34475347	34475473
CAT	chr11	34473623	34473754
CAT	chr11	34485651	34485782
CAT	chr11	34482797	34482936
CAT	chr11	34478211	34478364
CAT	chr11	34470738	34470910
CAT	chr11	34477557	34477749
CCR5	chr3	46414393	46415449
CDHR3	chr7	105667127	105667141
CDHR3	chr7	105668996	105669028
CDHR3	chr7	105603764	105603810
CDHR3	chr7	105671237	105671286
CDHR3	chr7	105635196	105635291
CDHR3	chr7	105624637	105624735
CDHR3	chr7	105656372	105656471
CDHR3	chr7	105655556	105655659
CDHR3	chr7	105636695	105636800
CDHR3	chr7	105641907	105642056
CDHR3	chr7	105621413	105621579
CDHR3	chr7	105653305	105653477
CDHR3	chr7	105660818	105660990
CDHR3	chr7	105664826	105665008
CDHR3	chr7	105644943	105645133
CDHR3	chr7	105615289	105615492
CDHR3	chr7	105658291	105658518
CDHR3	chr7	105662643	105662894
CDHR3	chr7	105672838	105673140
COPE	chr19	19010490	19010535
COPE	chr19	19016384	19016438
COPE	chr19	19023792	19023855
COPE	chr19	19011189	19011258

COPE	chr19	19016675	19016744
COPE	chr19	19010681	19010756
COPE	chr19	19015579	19015661
COPE	chr19	19021779	19021880
COPE	chr19	19030031	19030157
COPE	chr19	19017768	19017921
COPE	chr19	19014076	19014232
CYP2C9	chr10	96731860	96732002
CYP2C9	chr10	96745789	96745931
CYP2C9	chr10	96701948	96702103
CYP2C9	chr10	96707535	96707696
CYP2C9	chr10	96701614	96701777
CYP2C9	chr10	96748603	96748782
CYP2C9	chr10	96698439	96698607
CYP2C9	chr10	96708864	96709041
CYP2C9	chr10	96740939	96741127
CYP2D6	chr22	42523782	42523811
CYP2D6	chr22	42522852	42522994
CYP2D6	chr22	42523843	42523985
CYP2D6	chr22	42525034	42525187
CYP2D6	chr22	42524785	42524946
CYP2D6	chr22	42525739	42525911
CYP2D6	chr22	42522578	42522754
CYP2D6	chr22	42524175	42524352
CYP2D6	chr22	42526613	42526793
CYP2D6	chr22	42523448	42523664
CYP2E1	chr10	135347259	135347401
CYP2E1	chr10	135351254	135351396
CYP2E1	chr10	135345088	135345238
CYP2E1	chr10	135341984	135342144
CYP2E1	chr10	135345627	135345788
CYP2E1	chr10	135340899	135341076
CYP2E1	chr10	135346195	135346372
CYP2E1	chr10	135352283	135352465
CYP2E1	chr10	135350566	135350754
DCHS2	chr4	155244392	155244481
DCHS2	chr4	155249244	155249344
DCHS2	chr4	155191051	155191165
DCHS2	chr4	155163793	155163924
DCHS2	chr4	155160321	155160454
DCHS2	chr4	155226248	155226383
DCHS2	chr4	155176670	155176822
DCHS2	chr4	155243482	155243640
DCHS2	chr4	155250674	155250862
DCHS2	chr4	155298404	155298596

DCHS2	chr4	155225820	155226030
DCHS2	chr4	155180696	155180908
DCHS2	chr4	155287361	155287593
DCHS2	chr4	155256019	155256256
DCHS2	chr4	155236899	155237139
DCHS2	chr4	155161688	155161975
DCHS2	chr4	155241530	155242374
DCHS2	chr4	155219002	155219860
DCHS2	chr4	155253629	155254646
DCHS2	chr4	155410455	155412507
DCHS2	chr4	155155690	155158311
DOCK1	chr10	128776211	128776252
DOCK1	chr10	129178345	129178386
DOCK1	chr10	128594086	128594132
DOCK1	chr10	128780181	128780237
DOCK1	chr10	129152932	129152991
DOCK1	chr10	128904494	128904565
DOCK1	chr10	128806995	128807068
DOCK1	chr10	128824615	128824688
DOCK1	chr10	129137269	129137348
DOCK1	chr10	129207576	129207655
DOCK1	chr10	128797617	128797699
DOCK1	chr10	128768965	128769049
DOCK1	chr10	129217929	129218013
DOCK1	chr10	129209103	129209188
DOCK1	chr10	129172044	129172130
DOCK1	chr10	129213415	129213502
DOCK1	chr10	128840888	128840978
DOCK1	chr10	129201318	129201408
DOCK1	chr10	129179541	129179632
DOCK1	chr10	129249595	129249688
DOCK1	chr10	129055598	129055693
DOCK1	chr10	128925932	128926028
DOCK1	chr10	128785784	128785881
DOCK1	chr10	128835982	128836080
DOCK1	chr10	128822948	128823047
DOCK1	chr10	128841327	128841428
DOCK1	chr10	129141909	129142010
DOCK1	chr10	129046271	129046373
DOCK1	chr10	128829913	128830017
DOCK1	chr10	129202588	129202693
DOCK1	chr10	128908511	128908618
DOCK1	chr10	128859931	128860040
DOCK1	chr10	129183053	129183173
DOCK1	chr10	128821463	128821588

DOCK1	chr10	128816973	128817099
DOCK1	chr10	128923737	128923865
DOCK1	chr10	128850944	128851079
DOCK1	chr10	128795011	128795147
DOCK1	chr10	128798435	128798571
DOCK1	chr10	129224137	129224275
DOCK1	chr10	129207300	129207442
DOCK1	chr10	128810495	128810638
DOCK1	chr10	129231546	129231692
DOCK1	chr10	128788718	128788867
DOCK1	chr10	129160327	129160476
DOCK1	chr10	129245657	129245809
DOCK1	chr10	129242389	129242543
DOCK1	chr10	129172321	129172478
DOCK1	chr10	128796355	128796513
DOCK1	chr10	129216628	129216805
DOCK1	chr10	128830400	128830584
DOCK1	chr10	129237290	129237489
EGFL6	chrX	13587980	13588054
EGFL6	chrX	13637281	13637362
EGFL6	chrX	13612954	13613047
EGFL6	chrX	13641939	13642044
EGFL6	chrX	13651103	13651211
EGFL6	chrX	13607674	13607787
EGFL6	chrX	13618093	13618213
EGFL6	chrX	13621435	13621555
EGFL6	chrX	13626442	13626565
EGFL6	chrX	13624497	13624632
EGFL6	chrX	13645129	13645395
EGFL6	chrX	13635848	13636172
EML1	chr14	100276011	100276021
EML1	chr14	100352209	100352234
EML1	chr14	100204169	100204197
EML1	chr14	100357535	100357564
EML1	chr14	100341267	100341324
EML1	chr14	100259813	100259880
EML1	chr14	100384118	100384186
EML1	chr14	100364569	100364639
EML1	chr14	100316584	100316669
EML1	chr14	100402583	100402671
EML1	chr14	100387125	100387214
EML1	chr14	100373974	100374070
EML1	chr14	100404153	100404249
EML1	chr14	100402365	100402463
EML1	chr14	100376578	100376678

EML1	chr14	100367265	100367376
EML1	chr14	100406323	100406446
EML1	chr14	100380515	100380641
EML1	chr14	100360965	100361095
EML1	chr14	100405533	100405664
EML1	chr14	100380902	100381034
EML1	chr14	100331850	100331983
EML1	chr14	100344821	100344956
EML1	chr14	100375681	100375816
EML1	chr14	100363481	100363631
EML1	chr14	100377758	100377913
EML1	chr14	100317189	100317372
ETAA1	chr2	67626681	67626758
ETAA1	chr2	67629993	67630106
ETAA1	chr2	67637042	67637167
ETAA1	chr2	67626300	67626429
ETAA1	chr2	67624580	67624803
ETAA1	chr2	67630356	67632467
FLT1	chr13	28896592	28896614
FLT1	chr13	28963743	28963769
FLT1	chr13	29068916	29068980
FLT1	chr13	28973183	28973255
FLT1	chr13	28942717	28942800
FLT1	chr13	28882979	28883064
FLT1	chr13	28901598	28901687
FLT1	chr13	28880814	28880909
FLT1	chr13	29041657	29041754
FLT1	chr13	28896398	28896496
FLT1	chr13	28891634	28891734
FLT1	chr13	28908161	28908266
FLT1	chr13	28886129	28886235
FLT1	chr13	28919581	28919688
FLT1	chr13	28971096	28971205
FLT1	chr13	28893559	28893671
FLT1	chr13	28903751	28903865
FLT1	chr13	28979916	28980031
FLT1	chr13	29004186	29004304
FLT1	chr13	28895599	28895722
FLT1	chr13	29012357	29012482
FLT1	chr13	28931690	28931822
FLT1	chr13	28913304	28913437
FLT1	chr13	29007955	29008092
FLT1	chr13	28885726	28885869
FLT1	chr13	28959021	28959168
FLT1	chr13	28896926	28897083

FLT1	chr13	29001295	29001455
FLT1	chr13	29008194	29008357
FLT1	chr13	29001888	29002058
FLT1	chr13	29005272	29005447
FLT1	chr13	28877306	28877505
FLT1	chr13	29041039	29041266
FLT1	chr13	28963840	28964241
FZD5	chr2	208631708	208633463
GATA1	chrX	48651578	48651704
GATA1	chrX	48650729	48650875
GATA1	chrX	48649516	48649736
GATA1	chrX	48650250	48650628
GATA1	chrX	48652199	48652672
GATA2	chr3	128200661	128200787
GATA2	chr3	128202702	128202848
GATA2	chr3	128205645	128205874
GATA2	chr3	128199864	128200161
GATA2	chr3	128204569	128205211
GPM6B	chrX	13956625	13956629
GPM6B	chrX	13834962	13835023
GPM6B	chrX	13794356	13794422
GPM6B	chrX	13795470	13795544
GPM6B	chrX	13792665	13792743
GPM6B	chrX	13825768	13825888
GPM6B	chrX	13791002	13791149
GPM6B	chrX	13801483	13801640
GPM6B	chrX	13797936	13798108
GPM6B	chrX	13803740	13803927
HAO2	chr1	119923315	119923346
HAO2	chr1	119936407	119936460
HAO2	chr1	119935240	119935310
HAO2	chr1	119923700	119923839
HAO2	chr1	119925537	119925689
HAO2	chr1	119934732	119934891
HAO2	chr1	119929244	119929454
HAO2	chr1	119927398	119927676
HDAC1	chr1	32798617	32798642
HDAC1	chr1	32757770	32757819
HDAC1	chr1	32798301	32798350
HDAC1	chr1	32790079	32790154
HDAC1	chr1	32794668	32794761
HDAC1	chr1	32796178	32796287
HDAC1	chr1	32797074	32797183
HDAC1	chr1	32768221	32768334
HDAC1	chr1	32782265	32782383

HDAC1	chr1	32792539	32792678
HDAC1	chr1	32797276	32797407
HDAC1	chr1	32796368	32796509
HDAC1	chr1	32793136	32793278
HDAC1	chr1	32797690	32797843
HDAC2	chr6	114262224	114262252
HDAC2	chr6	114292020	114292072
HDAC2	chr6	114262871	114262929
HDAC2	chr6	114277782	114277857
HDAC2	chr6	114270331	114270424
HDAC2	chr6	114266525	114266634
HDAC2	chr6	114270142	114270251
HDAC2	chr6	114279812	114279930
HDAC2	chr6	114265443	114265574
HDAC2	chr6	114281069	114281203
HDAC2	chr6	114267171	114267312
HDAC2	chr6	114274440	114274582
HDAC2	chr6	114277160	114277315
HDAC2	chr6	114264514	114264670
HMOX1	chr22	35777166	35777189
HMOX1	chr22	35785856	35785956
HMOX1	chr22	35779098	35779219
HMOX1	chr22	35789460	35789588
HMOX1	chr22	35782677	35783169
KLF10	chr8	103665697	103665700
KLF10	chr8	103667793	103667829
KLF10	chr8	103664391	103664625
KLF10	chr8	103662362	103662619
KLF10	chr8	103663376	103664289
KLF4	chr9	110251448	110251453
KLF4	chr9	110251210	110251331
KLF4	chr9	110249308	110249473
KLF4	chr9	110248034	110248207
KLF4	chr9	110249575	110250548
KRT80	chr12	52565309	52565341
KRT80	chr12	52565441	52565497
KRT80	chr12	52574686	52574747
KRT80	chr12	52574296	52574392
KRT80	chr12	52566821	52566947
KRT80	chr12	52565184	52565306
KRT80	chr12	52567383	52567548
KRT80	chr12	52579162	52579371
KRT80	chr12	52565981	52566202
KRT80	chr12	52585386	52585686
LDB1	chr10	103879842	103879867

LDB1	chr10	103871012	103871057
LDB1	chr10	103870825	103870901
LDB1	chr10	103869356	103869440
LDB1	chr10	103870625	103870728
LDB1	chr10	103871190	103871293
LDB1	chr10	103869677	103869800
LDB1	chr10	103869140	103869264
LDB1	chr10	103868785	103868934
LDB1	chr10	103870285	103870458
LDB1	chr10	103867852	103868080
MAP3K5	chr6	136922159	136922180
MAP3K5	chr6	137026247	137026271
MAP3K5	chr6	136963657	136963707
MAP3K5	chr6	136878898	136878956
MAP3K5	chr6	136879937	136880014
MAP3K5	chr6	136958462	136958544
MAP3K5	chr6	136960680	136960776
MAP3K5	chr6	136901438	136901544
MAP3K5	chr6	136932419	136932525
MAP3K5	chr6	137017088	137017195
MAP3K5	chr6	136972121	136972229
MAP3K5	chr6	136882670	136882783
MAP3K5	chr6	136883640	136883753
MAP3K5	chr6	136990420	136990533
MAP3K5	chr6	136935296	136935424
MAP3K5	chr6	136943985	136944119
MAP3K5	chr6	136934257	136934394
MAP3K5	chr6	137041587	137041727
MAP3K5	chr6	136977444	136977598
MAP3K5	chr6	136926346	136926504
MAP3K5	chr6	136980356	136980516
MAP3K5	chr6	136913310	136913479
MAP3K5	chr6	137018356	137018525
MAP3K5	chr6	137015277	137015448
MAP3K5	chr6	136913574	136913752
MAP3K5	chr6	136922939	136923117
MAP3K5	chr6	136904692	136904878
MAP3K5	chr6	137019626	137019820
MAP3K5	chr6	136888768	136889012
MAP3K5	chr6	137112847	137113295
MAP7	chr6	136665368	136665376
MAP7	chr6	136787898	136787920
MAP7	chr6	136681118	136681169
MAP7	chr6	136680957	136681015
MAP7	chr6	136871479	136871546

MAP7	chr6	136732757	136732835
MAP7	chr6	136742838	136742937
MAP7	chr6	136681834	136681936
MAP7	chr6	136677829	136677932
MAP7	chr6	136704808	136704919
MAP7	chr6	136687464	136687577
MAP7	chr6	136698892	136699006
MAP7	chr6	136709530	136709648
MAP7	chr6	136846969	136847102
MAP7	chr6	136693638	136693787
MAP7	chr6	136710491	136710655
MAP7	chr6	136682142	136682317
MAP7	chr6	136666993	136667217
MAP7	chr6	136683587	136683840
MAP7	chr6	136686872	136687156
MAPK1	chr22	22127161	22127271
MAPK1	chr22	22123495	22123609
MAPK1	chr22	22142982	22143097
MAPK1	chr22	22153300	22153417
MAPK1	chr22	22221611	22221730
MAPK1	chr22	22142545	22142677
MAPK1	chr22	22161952	22162135
MAPK1	chr22	22160138	22160328
MAPK3	chr16	30128990	30129105
MAPK3	chr16	30129367	30129484
MAPK3	chr16	30127991	30128111
MAPK3	chr16	30128474	30128606
MAPK3	chr16	30128160	30128324
MAPK3	chr16	30134360	30134530
MAPK3	chr16	30129669	30129859
MAPK3	chr16	30133144	30133327
MARCH10	chr17	60782870	60782942
MARCH10	chr17	60879006	60879096
MARCH10	chr17	60799848	60799958
MARCH10	chr17	60788591	60788705
MARCH10	chr17	60824225	60824339
MARCH10	chr17	60865840	60865960
MARCH10	chr17	60778972	60779119
MARCH10	chr17	60821736	60821889
MARCH10	chr17	60802298	60802465
MARCH10	chr17	60837195	60837367
MARCH10	chr17	60827562	60827897
MARCH10	chr17	60813291	60814693
MET	chr7	116335810	116335853
MET	chr7	116417442	116417523

MET	chr7	116397490	116397593
MET	chr7	116422041	116422151
MET	chr7	116380003	116380138
MET	chr7	116397691	116397828
MET	chr7	116435708	116435845
MET	chr7	116411902	116412043
MET	chr7	116399390	116399544
MET	chr7	116411551	116411708
MET	chr7	116395408	116395569
MET	chr7	116398512	116398674
MET	chr7	116423357	116423523
MET	chr7	116380905	116381079
MET	chr7	116418829	116419011
MET	chr7	116371721	116371913
MET	chr7	116403103	116403322
MET	chr7	116409698	116409917
MET	chr7	116414934	116415165
MET	chr7	116435940	116436175
MET	chr7	116339124	116340338
MPO	chr17	56357726	56357820
MPO	chr17	56356883	56357007
MPO	chr17	56356657	56356787
MPO	chr17	56357965	56358119
MPO	chr17	56352902	56353063
MPO	chr17	56350108	56350279
MPO	chr17	56357199	56357375
MPO	chr17	56348019	56348224
MPO	chr17	56356368	56356575
MPO	chr17	56349015	56349253
MPO	chr17	56350774	56351030
MPO	chr17	56355187	56355506
MRM2	chr7	2280930	2280938
MRM2	chr7	2281796	2281804
MRM2	chr7	2279052	2279342
MRM2	chr7	2274759	2275199
MYBBP1A	chr17	4442237	4442270
MYBBP1A	chr17	4445758	4445827
MYBBP1A	chr17	4457313	4457388
MYBBP1A	chr17	4457489	4457573
MYBBP1A	chr17	4448553	4448640
MYBBP1A	chr17	4458155	4458251
MYBBP1A	chr17	4449140	4449237
MYBBP1A	chr17	4451252	4451352
MYBBP1A	chr17	4444757	4444859
MYBBP1A	chr17	4445078	4445186

MYBBP1A	chr17	4457104	4457212
MYBBP1A	chr17	4452626	4452737
MYBBP1A	chr17	4455174	4455292
MYBBP1A	chr17	4451818	4451944
MYBBP1A	chr17	4443642	4443779
MYBBP1A	chr17	4445910	4446036
MYBBP1A	chr17	4448320	4448470
MYBBP1A	chr17	4448904	4449056
MYBBP1A	chr17	4447783	4447941
MYBBP1A	chr17	4451437	4451605
MYBBP1A	chr17	4455419	4455587
MYBBP1A	chr17	4448045	4448216
MYBBP1A	chr17	4455745	4455921
MYBBP1A	chr17	4458421	4458619
MYBBP1A	chr17	4446207	4446460
MYBBP1A	chr17	4453352	4453648
MYBBP1A	chr17	4442712	4443262
N4BP2L2	chr13	33010521	33010542
N4BP2L2	chr13	33095509	33095586
N4BP2L2	chr13	33012788	33012874
N4BP2L2	chr13	33096318	33096407
N4BP2L2	chr13	33101544	33101669
N4BP2L2	chr13	33091941	33092140
N4BP2L2	chr13	33109905	33111164
N4BP2L2	chr13	33016524	33018263
NFKB1	chr4	103432089	103432106
NFKB1	chr4	103455001	103455042
NFKB1	chr4	103446668	103446714
NFKB1	chr4	103533200	103533267
NFKB1	chr4	103450992	103451071
NFKB1	chr4	103516048	103516138
NFKB1	chr4	103504022	103504114
NFKB1	chr4	103459014	103459113
NFKB1	chr4	103528805	103528908
NFKB1	chr4	103501691	103501796
NFKB1	chr4	103522051	103522166
NFKB1	chr4	103531731	103531856
NFKB1	chr4	103505838	103505977
NFKB1	chr4	103518676	103518818
NFKB1	chr4	103514581	103514725
NFKB1	chr4	103488143	103488292
NFKB1	chr4	103534581	103534738
NFKB1	chr4	103537590	103537748
NFKB1	chr4	103498032	103498196
NFKB1	chr4	103528306	103528476

NFKB1	chr4	103533590	103533763
NFKB1	chr4	103527652	103527854
NFKB1	chr4	103500009	103500196
NFKB1	chr4	103517294	103517489
NFKB2	chr10	104155716	104155737
NFKB2	chr10	104156205	104156246
NFKB2	chr10	104161205	104161275
NFKB2	chr10	104156009	104156091
NFKB2	chr10	104157968	104158054
NFKB2	chr10	104156481	104156580
NFKB2	chr10	104160703	104160806
NFKB2	chr10	104157737	104157842
NFKB2	chr10	104157058	104157165
NFKB2	chr10	104161804	104161916
NFKB2	chr10	104159836	104159951
NFKB2	chr10	104158495	104158621
NFKB2	chr10	104162008	104162130
NFKB2	chr10	104158141	104158280
NFKB2	chr10	104159333	104159475
NFKB2	chr10	104156660	104156812
NFKB2	chr10	104160936	104161088
NFKB2	chr10	104157283	104157442
NFKB2	chr10	104160411	104160581
NFKB2	chr10	104161501	104161674
NFKB2	chr10	104159044	104159254
NFKB2	chr10	104160034	104160248
NOM1	chr7	156761780	156761890
NOM1	chr7	156754844	156754955
NOM1	chr7	156756598	156756720
NOM1	chr7	156745167	156745292
NOM1	chr7	156759654	156759786
NOM1	chr7	156758963	156759096
NOM1	chr7	156755701	156755869
NOM1	chr7	156762222	156762394
NOM1	chr7	156746796	156746992
NOM1	chr7	156752544	156752868
NOM1	chr7	156742431	156743418
NOS1	chr12	117653116	117653129
NOS1	chr12	117696201	117696260
NOS1	chr12	117680431	117680510
NOS1	chr12	117701693	117701779
NOS1	chr12	117664487	117664575
NOS1	chr12	117723045	117723137
NOS1	chr12	117693740	117693842
NOS1	chr12	117705847	117705949

NOS1	chr12	117696830	117696935
NOS1	chr12	117691442	117691559
NOS1	chr12	117655850	117655969
NOS1	chr12	117662820	117662942
NOS1	chr12	117749270	117749397
NOS1	chr12	117728102	117728231
NOS1	chr12	117681101	117681240
NOS1	chr12	117715763	117715903
NOS1	chr12	117718529	117718671
NOS1	chr12	117698269	117698414
NOS1	chr12	117725878	117726024
NOS1	chr12	117660519	117660668
NOS1	chr12	117723908	117724071
NOS1	chr12	117669766	117669936
NOS1	chr12	117685152	117685327
NOS1	chr12	117710189	117710364
NOS1	chr12	117657879	117658074
NOS1	chr12	117703120	117703315
NOS1	chr12	117672369	117672563
NOS1	chr12	117665235	117665446
NOS1	chr12	117768149	117768874
NOS2	chr17	26096560	26096610
NOS2	chr17	26093535	26093614
NOS2	chr17	26100186	26100269
NOS2	chr17	26116629	26116714
NOS2	chr17	26088169	26088257
NOS2	chr17	26109040	26109132
NOS2	chr17	26105715	26105817
NOS2	chr17	26085906	26086101
NOS2	chr17	26084274	26084379
NOS2	chr17	26097938	26098043
NOS2	chr17	26120613	26120720
NOS2	chr17	26125725	26125835
NOS2	chr17	26087648	26087770
NOS2	chr17	26115834	26115957
NOS2	chr17	26094730	26094863
NOS2	chr17	26107792	26107932
NOS2	chr17	26108061	26108203
NOS2	chr17	26099333	26099478
NOS2	chr17	26087055	26087204
NOS2	chr17	26114703	26114852
NOS2	chr17	26109969	26110132
NOS2	chr17	26091006	26091170
NOS2	chr17	26096002	26096177
NOS2	chr17	26105907	26106082

NOS2	chr17	26092560	26092742
NOS2	chr17	26101282	26101477
NOS2	chr17	26089823	26090031
NOS3	chr7	150700398	150700488
NOS3	chr7	150700238	150700373
NOS3	chr7	150708875	150708927
NOS3	chr7	150703514	150703582
NOS3	chr7	150698631	150698705
NOS3	chr7	150706276	150706355
NOS3	chr7	150707986	150708074
NOS3	chr7	150695444	150695536
NOS3	chr7	150697585	150697687
NOS3	chr7	150699287	150699392
NOS3	chr7	150692290	150692402
NOS3	chr7	150703976	150704093
NOS3	chr7	150709438	150709560
NOS3	chr7	150706017	150706150
NOS3	chr7	150696033	150696173
NOS3	chr7	150695626	150695768
NOS3	chr7	150698908	150699053
NOS3	chr7	150693491	150693640
NOS3	chr7	150710318	150710467
NOS3	chr7	150690891	150691049
NOS3	chr7	150711095	150711254
NOS3	chr7	150693850	150694013
NOS3	chr7	150707202	150707375
NOS3	chr7	150696277	150696452
NOS3	chr7	150704189	150704364
NOS3	chr7	150698318	150698513
NOS3	chr7	150706485	150706673
NOS3	chr7	150710811	150711006
NOS3	chr7	150707684	150707895
OPRL1	chr20	62724073	62724306
OPRL1	chr20	62729154	62729510
OPRL1	chr20	62729628	62730149
OR51B5	chr11	5363818	5364754
OR51B6	chr11	5372737	5373673
PAPLN	chr14	73706486	73706540
PAPLN	chr14	73712343	73712404
PAPLN	chr14	73718193	73718274
PAPLN	chr14	73725686	73725767
PAPLN	chr14	73712780	73712883
PAPLN	chr14	73733430	73733539
PAPLN	chr14	73711351	73711467
PAPLN	chr14	73727416	73727537

PAPLN	chr14	73717614	73717738
PAPLN	chr14	73718725	73718849
PAPLN	chr14	73730918	73731043
PAPLN	chr14	73719356	73719483
PAPLN	chr14	73716691	73716822
PAPLN	chr14	73730352	73730490
PAPLN	chr14	73732077	73732215
PAPLN	chr14	73727863	73728002
PAPLN	chr14	73731295	73731434
PAPLN	chr14	73733208	73733336
PAPLN	chr14	73721584	73721726
PAPLN	chr14	73735264	73735431
PAPLN	chr14	73739202	73739369
PAPLN	chr14	73718371	73718544
PAPLN	chr14	73721220	73721403
PAPLN	chr14	73720461	73720669
PAPLN	chr14	73725976	73726253
PAPLN	chr14	73729057	73729535
PDE7B	chr6	136173136	136173157
PDE7B	chr6	136268601	136268662
PDE7B	chr6	136470223	136470288
PDE7B	chr6	136508173	136508254
PDE7B	chr6	136429868	136429952
PDE7B	chr6	136494934	136495026
PDE7B	chr6	136472297	136472393
PDE7B	chr6	136502364	136502461
PDE7B	chr6	136475212	136475313
PDE7B	chr6	136500134	136500279
PDE7B	chr6	136468488	136468640
PDE7B	chr6	136512751	136512975
PDE7B	chr6	136359428	136359666
PDE7B	chr6	136476764	136477100
PHC3	chr3	169899489	169899503
PHC4	chr3	169846127	169846175
PHC5	chr3	169863210	169863309
PHC6	chr3	169824619	169824734
PHC7	chr3	169889121	169889238
PHC8	chr3	169820589	169820722
PHC9	chr3	169815020	169815172
PHC10	chr3	169840378	169840532
PHC11	chr3	169890344	169890500
PHC12	chr3	169866873	169867032
PHC13	chr3	169831147	169831307
PHC14	chr3	169896560	169896726
PHC15	chr3	169820266	169820498

PHC16	chr3	169854206	169854453
PHC17	chr3	169834923	169835264
PHC18	chr3	169846471	169847340
PKD1L1	chr7	47780364	47780511
PKD1L1	chr7	47814743	47814764
PKD1L1	chr7	47987993	47988037
PKD1L1	chr7	47873936	47873983
PKD1L1	chr7	47904813	47904870
PKD1L1	chr7	47924195	47924255
PKD1L1	chr7	47874564	47874637
PKD1L1	chr7	47920325	47920403
PKD1L1	chr7	47853528	47853612
PKD1L1	chr7	47884592	47884676
PKD1L1	chr7	47869637	47869722
PKD1L1	chr7	47892724	47892825
PKD1L1	chr7	47880058	47880165
PKD1L1	chr7	47860673	47860784
PKD1L1	chr7	47874730	47874841
PKD1L1	chr7	47894752	47894863
PKD1L1	chr7	47913496	47913608
PKD1L1	chr7	47976442	47976555
PKD1L1	chr7	47982992	47983108
PKD1L1	chr7	47866947	47867066
PKD1L1	chr7	47945439	47945559
PKD1L1	chr7	47915694	47915815
PKD1L1	chr7	47971532	47971653
PKD1L1	chr7	47854931	47855055
PKD1L1	chr7	47979789	47979914
PKD1L1	chr7	47941975	47942108
PKD1L1	chr7	47842807	47842942
PKD1L1	chr7	47870814	47870950
PKD1L1	chr7	47849037	47849175
PKD1L1	chr7	47847844	47847985
PKD1L1	chr7	47917086	47917229
PKD1L1	chr7	47894479	47894637
PKD1L1	chr7	47927587	47927748
PKD1L1	chr7	47835586	47835748
PKD1L1	chr7	47872687	47872850
PKD1L1	chr7	47955028	47955196
PKD1L1	chr7	47944753	47944922
PKD1L1	chr7	47832224	47832395
PKD1L1	chr7	47852718	47852891
PKD1L1	chr7	47876518	47876692
PKD1L1	chr7	47947673	47947847
PKD1L1	chr7	47869022	47869199

PKD1L1	chr7	47921506	47921683
PKD1L1	chr7	47886476	47886669
PKD1L1	chr7	47933474	47933656
PKD1L1	chr7	47906016	47906212
PKD1L1	chr7	47937584	47937791
PKD1L1	chr7	47882559	47882767
PKD1L1	chr7	47898270	47898483
PKD1L1	chr7	47879043	47879260
PKD1L1	chr7	47970700	47970918
PKD1L1	chr7	47930139	47930361
PKD1L1	chr7	47897202	47897430
PKD1L1	chr7	47840246	47840477
PKD1L1	chr7	47851414	47851649
PKD1L1	chr7	47943974	47944214
PKD1L1	chr7	47968800	47969123
PKD1L1	chr7	47925283	47925652
RHPN2	chr19	33555689	33555758
RHPN2	chr19	33512476	33512552
RHPN2	chr19	33484882	33484959
RHPN2	chr19	33503552	33503630
RHPN2	chr19	33535154	33535270
RHPN2	chr19	33490491	33490611
RHPN2	chr19	33502584	33502709
RHPN2	chr19	33517409	33517538
RHPN2	chr19	33482728	33482875
RHPN2	chr19	33481419	33481575
RHPN2	chr19	33493152	33493309
RHPN2	chr19	33498919	33499086
RHPN2	chr19	33486931	33487126
RHPN2	chr19	33493718	33493906
RHPN2	chr19	33470904	33471162
RNF113B	chr13	98828419	98828430
RNF113B	chr13	98828535	98829490
RSPH3	chr6	159399291	159399378
RSPH3	chr6	159414870	159414958
RSPH3	chr6	159407311	159407453
RSPH3	chr6	159404693	159404839
RSPH3	chr6	159401805	159401968
RSPH3	chr6	159403516	159403720
RSPH3	chr6	159398572	159398880
RSPH3	chr6	159420466	159421008
SALL2	chr14	21994194	21994261
SALL2	chr14	22004982	22005055
SALL2	chr14	21990144	21990378
SALL2	chr14	21990840	21993788

SAR1A	chr10	71914843	71914880
SAR1A	chr10	71921613	71921671
SAR1A	chr10	71920759	71920825
SAR1A	chr10	71912234	71912348
SAR1A	chr10	71917504	71917623
SAR1A	chr10	71921374	71921494
SAR1A	chr10	71913593	71913725
SEC31B	chr10	102249459	102249518
SEC31B	chr10	102247783	102247855
SEC31B	chr10	102259280	102259355
SEC31B	chr10	102276630	102276709
SEC31B	chr10	102268763	102268859
SEC31B	chr10	102258916	102259015
SEC31B	chr10	102258461	102258561
SEC31B	chr10	102266077	102266177
SEC31B	chr10	102248614	102248731
SEC31B	chr10	102275852	102275976
SEC31B	chr10	102257423	102257550
SEC31B	chr10	102265117	102265252
SEC31B	chr10	102267181	102267324
SEC31B	chr10	102267664	102267808
SEC31B	chr10	102256891	102257037
SEC31B	chr10	102249008	102249155
SEC31B	chr10	102255141	102255303
SEC31B	chr10	102265796	102265958
SEC31B	chr10	102256014	102256188
SEC31B	chr10	102247375	102247552
SEC31B	chr10	102250462	102250640
SEC31B	chr10	102257785	102257964
SEC31B	chr10	102269072	102269268
SEC31B	chr10	102262010	102262241
SEC31B	chr10	102249764	102250079
SESN1	chr6	109323480	109323546
SESN1	chr6	109308749	109308833
SESN1	chr6	109330561	109330663
SESN1	chr6	109313989	109314102
SESN1	chr6	109309745	109309890
SESN1	chr6	109315664	109315812
SESN1	chr6	109311847	109312038
SESN1	chr6	109322490	109322691
SESN1	chr6	109321693	109321876
SESN1	chr6	109319715	109319958
SESN1	chr6	109414997	109415276
SIN3A	chr15	75699395	75699485
SIN3A	chr15	75673953	75674046

SIN3A	chr15	75672969	75673064
SIN3A	chr15	75706545	75706652
SIN3A	chr15	75692380	75692497
SIN3A	chr15	75694192	75694311
SIN3A	chr15	75702474	75702627
SIN3A	chr15	75702176	75702332
SIN3A	chr15	75681992	75682162
SIN3A	chr15	75676604	75676778
SIN3A	chr15	75722527	75722716
SIN3A	chr15	75687020	75687204
SIN3A	chr15	75668005	75668213
SIN3A	chr15	75693070	75693281
SIN3A	chr15	75664322	75664550
SIN3A	chr15	75688598	75688837
SIN3A	chr15	75703832	75704084
SIN3A	chr15	75714885	75715164
SIN3A	chr15	75705103	75705386
SIN3A	chr15	75684582	75685156
SLC14A1	chr18	43328340	43328390
SLC14A1	chr18	43332142	43332224
SLC14A1	chr18	43314238	43314367
SLC14A1	chr18	43319492	43319627
SLC14A1	chr18	43307236	43307383
SLC14A1	chr18	43319127	43319275
SLC14A1	chr18	43329742	43329913
SLC14A1	chr18	43310264	43310436
SLC14A1	chr18	43316420	43316613
SLC14A1	chr18	43310979	43311169
SLC14A2	chr18	43223096	43223146
SLC14A2	chr18	43258939	43258989
SLC14A2	chr18	43246101	43246184
SLC14A2	chr18	43243749	43243872
SLC14A2	chr18	43212314	43212443
SLC14A2	chr18	43248313	43248442
SLC14A2	chr18	43221173	43221308
SLC14A2	chr18	43253647	43253782
SLC14A2	chr18	43219710	43219858
SLC14A2	chr18	43252864	43253012
SLC14A2	chr18	43204629	43204779
SLC14A2	chr18	43246899	43247059
SLC14A2	chr18	43223950	43224125
SLC14A2	chr18	43205647	43205828
SLC14A2	chr18	43216954	43217147
SLC14A2	chr18	43206922	43207112
SLC14A2	chr18	43247797	43247987

SLC14A2	chr18	43262283	43262481
SLC14A2	chr18	43249270	43249463
SLC22A4	chr5	131679452	131679525
SLC22A4	chr5	131667448	131667543
SLC22A4	chr5	131647853	131647957
SLC22A4	chr5	131662969	131663096
SLC22A4	chr5	131676257	131676393
SLC22A4	chr5	131649306	131649461
SLC22A4	chr5	131657876	131658048
SLC22A4	chr5	131671510	131671693
SLC22A4	chr5	131670410	131670625
SLC22A4	chr5	131630309	131630702
SLC22A5	chr5	131713855	131713927
SLC22A5	chr5	131729876	131729961
SLC22A5	chr5	131724612	131724713
SLC22A5	chr5	131714069	131714173
SLC22A5	chr5	131722716	131722843
SLC22A5	chr5	131729367	131729503
SLC22A5	chr5	131719838	131719993
SLC22A5	chr5	131721019	131721191
SLC22A5	chr5	131728124	131728307
SLC22A5	chr5	131726381	131726596
SLC22A5	chr5	131705664	131706057
SLCO1A2	chr12	21472305	21472359
SLCO1A2	chr12	21487521	21487581
SLCO1A2	chr12	21428293	21428358
SLCO1A2	chr12	21454104	21454203
SLCO1A2	chr12	21459815	21459922
SLCO1A2	chr12	21427402	21427520
SLCO1A2	chr12	21467482	21467615
SLCO1A2	chr12	21471715	21471857
SLCO1A2	chr12	21457360	21457507
SLCO1A2	chr12	21450337	21450502
SLCO1A2	chr12	21446878	21447044
SLCO1A2	chr12	21445097	21445270
SLCO1A2	chr12	21448530	21448726
SLCO1A2	chr12	21422484	21422701
SLCO1A2	chr12	21453281	21453503
SLCO1B1	chr12	21375233	21375298
SLCO1B1	chr12	21331509	21331656
SLCO1B1	chr12	21294508	21294592
SLCO1B1	chr12	21331855	21331954
SLCO1B1	chr12	21377655	21377773
SLCO1B1	chr12	21329709	21329831
SLCO1B1	chr12	21327510	21327643

SLCO1B1	chr12	21325583	21325725
SLCO1B1	chr12	21353441	21353606
SLCO1B1	chr12	21358801	21358967
SLCO1B1	chr12	21355424	21355620
SLCO1B1	chr12	21370052	21370237
SLCO1B1	chr12	21391912	21392120
SLCO1B1	chr12	21349879	21350122
SLCO1B3	chr12	21051369	21051434
SLCO1B3	chr12	20968672	20968756
SLCO1B3	chr12	21015689	21015788
SLCO1B3	chr12	21054833	21054933
SLCO1B3	chr12	21007961	21008103
SLCO1B3	chr12	21054283	21054401
SLCO1B3	chr12	21013950	21014072
SLCO1B3	chr12	21011372	21011505
SLCO1B3	chr12	21015345	21015492
SLCO1B3	chr12	21030705	21030870
SLCO1B3	chr12	21033788	21033954
SLCO1B3	chr12	21036351	21036536
SLCO1B3	chr12	21032369	21032565
SLCO1B3	chr12	21068937	21069178
SLCO1B3	chr12	21028168	21028411
SOX6	chr11	16424204	16424209
SOX6	chr11	16205431	16205500
SOX6	chr11	16119154	16119234
SOX6	chr11	16256127	16256217
SOX6	chr11	16036487	16036596
SOX6	chr11	16133348	16133469
SOX6	chr11	16117541	16117664
SOX6	chr11	16077297	16077447
SOX6	chr11	16208328	16208501
SOX6	chr11	16068059	16068247
SOX6	chr11	16339991	16340199
SOX6	chr11	16007749	16007966
SOX6	chr11	16071258	16071484
SOX6	chr11	16010542	16010776
SOX6	chr11	16362556	16362798
SOX6	chr11	15994357	15994658
SRY	chrY	2655032	2655644
SUDS3	chr12	118827731	118827751
SUDS3	chr12	118839787	118839809
SUDS3	chr12	118821816	118821872
SUDS3	chr12	118839563	118839625
SUDS3	chr12	118817966	118818036
SUDS3	chr12	118823918	118823990

SUDS3	chr12	118848877	118848962
SUDS3	chr12	118838463	118838559
SUDS3	chr12	118852139	118852235
SUDS3	chr12	118841216	118841322
SUDS3	chr12	118814496	118814638
SUDS3	chr12	118828930	118829087
TAL1	chr1	47689675	47689770
TAL1	chr1	47691114	47691560
TAL1	chr1	47685394	47685846
TOX	chr8	59720308	59720342
TOX	chr8	59872501	59872567
TOX	chr8	59739380	59739461
TOX	chr8	60031444	60031546
TOX	chr8	59720676	59720828
TOX	chr8	59750639	59750870
TOX	chr8	59851860	59852103
TOX	chr8	59764082	59764364
TOX	chr8	59727896	59728283
TTLL10	chr1	1117120	1117195
TTLL10	chr1	1115036	1115117
TTLL10	chr1	1117740	1117826
TTLL10	chr1	1132472	1132566
TTLL10	chr1	1131979	1132096
TTLL10	chr1	1114595	1114713
TTLL10	chr1	1115862	1115981
TTLL10	chr1	1116110	1116240
TTLL10	chr1	1120348	1120519
TTLL10	chr1	1118255	1118427
TTLL10	chr1	1119299	1119471
TTLL10	chr1	1115413	1115720
TTLL10	chr1	1132817	1133224
UGT1A1	chr2	234678176	234678204
UGT1A1	chr2	234676494	234676582
UGT1A1	chr2	234675679	234675811
UGT1A1	chr2	234676865	234677085
UGT1A1	chr2	234680907	234681202
UGT1A1	chr2	234668933	234669797
